# The Contribution of Silk Fibroin in Biomedical Engineering

**DOI:** 10.3390/insects13030286

**Published:** 2022-03-14

**Authors:** Cristian Lujerdean, Gabriela-Maria Baci, Alexandra-Antonia Cucu, Daniel Severus Dezmirean

**Affiliations:** Faculty of Animal Science and Biotechnology, University of Animal Sciences and Veterinary Medicine Cluj-Napoca, 400372 Cluj-Napoca, Romania; antonia.cucu@usamvcluj.ro (A.-A.C.); ddezmirean@usamvcluj.ro (D.S.D.)

**Keywords:** silk fibroin, biocompatibility, green material, biomaterial, 3D scaffolds, cancer therapy

## Abstract

**Simple Summary:**

In the medical area and beyond one of the most important biomaterials is the silk fibroin (SF) produced by the *Bombyx mori* L. silkworm. This outstanding biopolymer has received great attention from researchers due to its unique properties. Among them, the most important characteristic of SF is the high level of biocompatibility with the human organism. The biocompatibility, high mechanical strength, biodegradability and the biologically active properties have put SF in the spotlight, and thus numerous biomaterials have been developed. Furthermore, by using genetic engineering, biomaterials have been obtained that exhibit enhanced properties. In a wide range of studies, SF was used in order to develop sponges, hydrogels, nanospheres and films. By using SF-based biomaterials, tremendous progress has been made in tissue engineering and cancer therapy. In the specialized literature, various methods have been described regarding both extraction and processing of SF as a functional material. Moreover, SF-based biomaterials have been successfully obtained by using ecological methods of processing. Therefore, SF is considered to be the foremost green material.

**Abstract:**

Silk fibroin (SF) is a natural protein (biopolymer) extracted from the cocoons of *Bombyx mori* L. (silkworm). It has many properties of interest in the field of biotechnology, the most important being biodegradability, biocompatibility and robust mechanical strength with high tensile strength. SF is usually dissolved in water-based solvents and can be easily reconstructed into a variety of material formats, including films, mats, hydrogels, and sponges, by various fabrication techniques (spin coating, electrospinning, freeze-drying, and physical or chemical crosslinking). Furthermore, SF is a feasible material used in many biomedical applications, including tissue engineering (3D scaffolds, wounds dressing), cancer therapy (mimicking the tumor microenvironment), controlled drug delivery (SF-based complexes), and bone, eye and skin regeneration. In this review, we describe the structure, composition, general properties, and structure–properties relationship of SF. In addition, the main methods used for ecological extraction and processing of SF that make it a green material are discussed. Lastly, technological advances in the use of SF-based materials are addressed, especially in healthcare applications such as tissue engineering and cancer therapeutics.

## 1. Introduction

In the past years, research in the biomaterials field has expanded considerably through unprecedented progress and through the introduction of a significant number of innovative materials [1]. As a result, biomaterials currently have a widespread application in medicine due to their specific combination of resistance and biological compatibility. Their ability to induce tissue and organ regeneration has made it an extraordinary option for therapeutic treatments [2,3]. Moreover, modern medicine has been increasingly using various synthetic or natural materials (generally called “biomaterials”) for improving the quality of life and longevity of human beings. A biomaterial is intended to interact with biological systems to evaluate, treat, enlarge, or replace any tissue, organ, or function of the body. Numerous biomaterials are used in the human body, including metals, ceramics, and synthetic and natural polymers [4]. Of these, natural polymers (biopolymers) form the largest class of biomaterials used in medicine, possessing a number of unique properties covering a wide variety of clinical applications, especially in regenerative medicine and tissue engineering [5]. At the same time, they are able to mimic the structure of human tissue due to their physical and chemical resemblance, and most importantly, they are less toxic to native healthy tissue and more biocompatible compared to most synthetic materials [6,7].

Among biopolymers, silk fibroin (SF) has attracted more and more attention from life scientists all over the world from various fields such as chemistry, physics, engineering, biology, and last but not least, medicine. This protein is regarded as a remarkable bioactive material for tissue engineering applications due to its biocompatibility, biodegradability, and tunability [8], and it is studied in the production of 3D scaffolds mixed with various materials (natural or synthetic polymers, drugs, nanoparticles, growth factors, bioactive molecules, etc.) that together promote wound healing [9,10,11,12,13,14]. In addition, it can be processed in a wide range of formats, including sponges, wafers, gauze, particles, hydrogels, fibers, and films [15], being feasible for many other applications. SF as a biomaterial displays remarkable biological and mechanical properties such as controlled biodegradability, high biocompatibility, optical transparency, flexibility, mechanical resistance and processability. It is one of the main natural fibers produced by the *Bombyx mori* L. silkworm, and the purification and further processing of this protein as regenerated silk fibroin (RSF) can be obtained by eco-friendly methods that reduce or remove the need for chemical solvents and energy-intensive equipment. These eco-properties combined with a water-based extraction and purification process make SF a promising material for sustainable manufacturing, enabling it to partially replace synthetic, plastic-based and non-biodegradable material [16,17].

**Figure 1:**
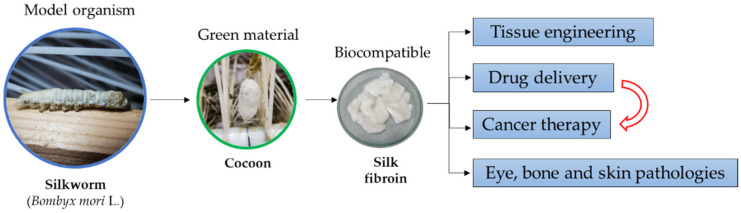
Schematic representation of SF main applications.

In recent years, numerous studies have been conducted focusing on the widespread use of SF as a functional biomaterial in the biomedical field. This review is expected to provide information on superior mechanical and biological properties of SF, the processing methods as a green material, recent advances in tissue engineering where SF plays a major role in mimicking the microenvironment of cells, and the importance of SF-based biomaterials used in drug delivery and cancer therapy. Figure 1 presents a schematic image of the main SF application.

## 2. SF—Overview

Silks, as natural polymers, play a pivotal role for insects, being involved in the processes of survival and reproduction. Owning remarkable properties, silk has been the subject of various studies for more than a century [18,19], and it is appreciated by the scientific community in the field of biology, biotechnology and materials [20]. The main sources for this outstanding natural protein biopolymer are the spider (*Nephila clavipes*) and the silkworm (*B. mori*). *N. clavipes* exhibits certain behaviors that do not allow for the spider’s large-scale harvesting [21]. Conversely, for more than 5000 years, silkworms have been domesticated and are currently being reared in numerous countries, such as China, India, and Japan, etc. As anticipated, the silk obtained from *B. mori* has been the most explored. Keeping this in mind, silkworms are the most feasible biological system in the context of silk production [22,23,24]. In the past, the silkworms were reared in order to obtain silk for textile applications; however, currently, silk receives considerable attention for its various purposes in life science areas [25]. 

The silk produced by *B. mori* is composed of two proteins, fibroin and sericin. Of the two proteins, fibroin (~400 kDa) is the core peptide. The silk fibers involve two fibroin filaments banded together by sericin, which has a glue-like activity [26,27]. Every fibroin molecule involves two chains, the fibroin heavy chain (FibH) and the fibroin light chain (FibL). Between the two chains, there is a disulfide bond through which the two proteins are linked. Besides FibH and FibL, SF also includes one more protein, namely fibrohexamerin/P25. The FibH chain contains multiple repetitive sequences that are involved in the development of anti-parallel structures. However, the FibL chain contains non-repetitive sequences that are responsible for the chain’s hydrophilicity and fibroin’s elasticity [28]. The molar ratio of FibH, FibL and P25 in fibroin molecules is 6:6:1 [29]. In a recent study, it was shown that the genes that encode the three fibroin proteins are directly regulated by the promoter-interacting proteins. Particularly, their data revealed that there are numerous proteins of this type that impact the ribosome and metabolism pathways [30]. The architecture and mechanical strength of SF assembled by *B. mori* are defined by the protein’s amino acid structure and sequence. With regard to the fibroin’s primary structure, three main amino acids are described, namely glycine, alanine and serine [31]. However, tyrosine, valine, histidine and tryptophan are also involved in fibroin’s structure, but in smaller amounts. SF possesses crystalline domains, but it also contains amorphous domains [32]. This outstanding protein, SF, displays three distinct polymorphs (I, II, III) [26,33]. Silk I and silk II are the crystalline forms of fibroin. The first one represents the structure of fibroin before spinning and belongs to the orthorhombic crystal system, and the last one is the protein’s solid state obtained after the spinning process, as part of the monoclinic crystal system. However, by treating silk I with potassium phosphate, it will be converted to the second polymorph [28]. 

The silk gland is the silk producing unit, with fibroin being assembled in the posterior silk gland (PSG) and sericin synthesized in the middle silk gland. After being synthetized, the proteins form an aqueous solution and are deposited into the silk gland lumen. When the process of spinning occurs, the proteins proceed through the anterior silk gland (ASG) duct [34].

## 3. SF Key Properties

**Figure 2:**
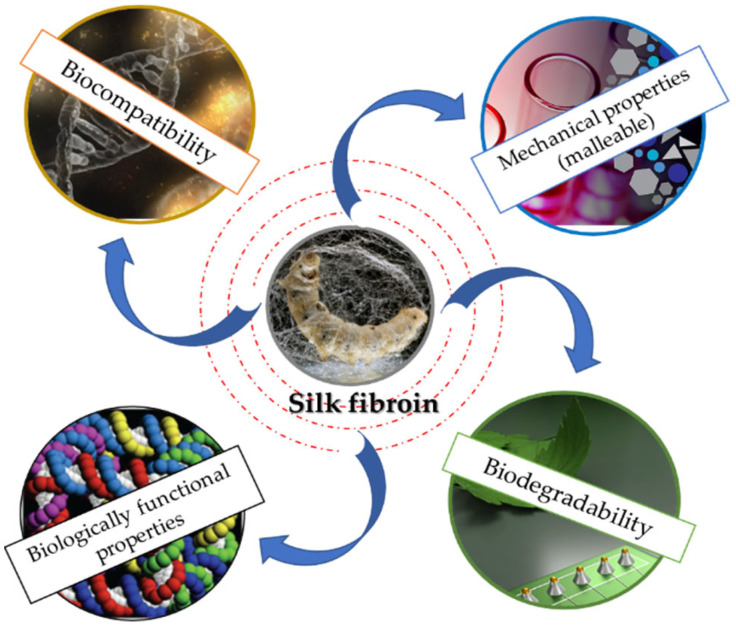
Schematic representation of SF properties.

SF exhibits numerous properties that convert this outstanding polymer into one of the most appreciated biomaterials (Figure 2). Currently, there are various processing forms for SF: scaffolds, sponges, or films, etc. [35,36,37,38,39,40]. It has been shown that the biocompatibility feature is influenced by the purification method and the purification process. Numerous studies have reported that SF’s biocompatibility is maximized after the removal of the sericin [41,42]. However, by using sericin as a biomaterial, minimal inflammatory responses have been reported, indicating that the immune system is active by the combination of the two proteins [43].

### 3.1. Biocompatibility

The applicability of SF in the medical field dates to the 19th century. It has been intensively used as sutures due to its high biocompatibility with the human organism. There are numerous reports that have confirmed this extraordinary property of SF [44,45,46,47,48,49,50,51]. 

Burn wounds represent one of the most severe categories of injuries; they cause massive loss of life worldwide. When a burn injury occurs, the patient exhibits great vulnerability to numerous photogenic bacteria [52]. Through reoccurring bacterial infections, the healing process can be severely delayed. In order to prevent bacterial infections, the scientific community is currently focused on developing advanced dressings that can exhibit great protection against certain pathogens [53]. Yin et al. (2022) developed SF-based hydrogels and incorporated rhein into its structure, due to the fact that rhein possesses certain antibacterial and anti-inflammatory properties. After developing the target biomaterial, the authors determined the hydrogel biocompatibility by evaluating the hemolysis ratio observed after the blood was exposed to the hydrogel for 1 h. Their results showed favorable biocompatibility. Furthermore, the authors used mice as an animal model to demonstrate in vivo the protective activity against infections, thus highlighting the feasibility of using SF-based dressings for accelerated tissue healing [54].

One of the most common neurodegenerative disorders is Alzheimer’s disease. In Alzheimer’s disease, the hippocampus is the most vulnerable are of the brain [55,56]. Tang et al. (2008) [57] performed an in vitro study in order to investigate the biocompatibility of hippocampal neurons with SF biomaterials. The authors removed the sericin from the silk fibers and developed an SF-based substrate in order to use it to culture the hippocampal neurons. With regard to central nervous system therapy, one of the most used biomaterials is polyglycolic acid (PGA). Their findings showed that by using SF as a biomaterial, similar results with PGA fibers were observed, indicating that, in vitro, fibroin displays the essential conditions for a biomaterial [57]. Another in vitro study evaluated the biocompatibility of SF fibers and extract fluid, respectively, with rat dorsal root ganglia and Schwann cells derived from sciatic nerves. The data indicated that SF did not exhibit any cytotoxicity against either biological specimens. This progress is extremely important for the development of artificial nerve grafts [46]. 

Skin represents the largest and one of the most important organs of humans. It has numerous functions; thus, the wound healing process plays a pivotal role in human health [58]. In this direction, Zhang et al. (2017) [59] explored the effectiveness of using SF-based films in the skin repair process by using rabbits and porcine as animal models. This study highlighted the high biocompatibility and the effectiveness of SF as a biomaterial [59]. 

Macrophages play a key role in the regulation of the host’s immune response. An ideal biomaterial should exhibit great biocompatibility; therefore, it should not induce appreciable macrophage response. The macrophage response had to be evaluated in order to confirm the biocompatibility of each biomaterial. In this direction, a research group investigated the macrophage response by adhering SF films to L929 murine fibroblasts. By performing this experiment, extremely low macrophage activation was observed, suggesting the high biocompatibility of SF as a biomaterial [60].

Moreover, in a recent study, SF produced by *B. mori* was used to develop bioink for three-dimensional (3D) bioprinting. Bioink is in the spotlight due to its applicability in the area of tissue and organ engineering. In this study, the authors used glycidyl methacrylate to chemically modify the SF in order to obtain the bioink. They acquired SF-based hydrogels by employing 3D bioprinting and demonstrated its great biocompatibility by using NIH/3T3 fibroblasts. Furthermore, in order to investigate the long-term biocompatibility of this specific biomaterial, the SF hydrogels were used for printing various complex biological structures, such as cartilaginous trachea or lungs. This research highlighted the feasibility of SF-based hydrogels for 3D bioprinting [61].

### 3.2. Biodegradability

Biodegradation is the process of disruption of natural polymers into numerous small-scale compounds. Regarding the applicability of biomaterials in the medical area, one of the most key requirements is biodegradability. Due to SF’s biodegradable behavior, this natural biopolymer, together with chitosan or collagen, represents one of the most used biomaterials for tissue engineering applications and beyond. There are three main proteolytic enzymes that degrade SF, namely chymotrypsin, carboxylase and actinase. After the process of biodegradation occurs, both the structure and the molecular mass of SF suffer notable changes [62]. 

SF’s degradation behavior is impacted by several factors. The most important factor that influences the degradation rate of SF-based biomaterials is pore size [63,64,65]. By comparing the degradation rate of SF-based scaffolds with large and small pore sizes, it has been demonstrated that the biomaterials that had large pores degraded faster than the ones with smaller pores. Conversely, scaffolds with higher pore density deteriorated slowly compared to the ones with lower pore density [66].

In a recent study, Sun et al. (2022) [67] developed SF sponges by using the ice-cold-induced phase separation-based method. Moreover, the cryo-sponges contained exosomes in order to obtain controlled delivery of the specific extracellular vesicles. Their data showed that the encapsulated exosomes maintained their characteristics, and for two months, the vesicles remained undigested. Furthermore, in vivo assessments showed that the sustained release promoted cell migration activity, but also stimulated the development of novel blood vessels. Moreover, their results showed that the SF sponges’ biodegradability rate was correlated with the fibroin concentrations [67]. This study highlights the great potential of SF as a biodegradable biomaterial for sustained drug delivery. 

Catto et al. (2015) [68] successfully designed SF-based tubular vascular grafts. They tested, in vitro, the degradation behavior of SF tubes and confirmed the fact that the enzymatic activity impacted the SF’s crystallinity [68]. In another study, SF was associated with chitin in order to develop scaffolds that harbor silver nanoparticles that exhibit antimicrobial activity for wound dressings. The authors demonstrated the antimicrobial effect of nanocomposite scaffolds against several bacteria. The biodegradability of the obtained biomaterial was tested in vitro by using lysozyme. Their data showed that scaffolds exhibit great biodegradable behavior; moreover, the degradation products did not exhibit cytotoxicity [69].

Fan et al. (2009) [70] used pigs as an experimental model in order to evaluate the potential of employing SF scaffolds for clinical applications. By using an SF scaffold in pigs, the authors aimed to regenerate one of the key players for the strength of the knee joint, namely the anterior cruciate ligament. Furthermore, they developed mesenchymal stem cells-seeded SF-based scaffolds and implanted them in the experimental animal model. After 24 weeks, the mesenchymal stem cells displayed fibroblast morphology; moreover, great scaffold degradation was perceived. Conversely, after 24 weeks, the ultimate tensile load of the repaired anterior cruciate ligament was sustained [70].

In another study, 3D porous SF scaffolds were obtained by using two different processing methods, aqueous or organic solvent-based techniques. Additionally, the impact of several processing variables on SF scaffold behavior was investigated; specifically, the authors explored how the SF concentration and scaffolds’ pore size influenced short- and long-term in vivo behavior of this biomaterial. As an animal experimental model, two types of rats were used, nude and Lewis. The aqueous-derived scaffolds compared with the organic solvent (hexafluoroisopropanol)-derived, exhibited more homogeneous degradability. However, the hexafluoroisopropanol-derived scaffolds were more impacted by the SF concentration and pore size; more specifically, a slower degradation rate was observed by using a higher SF concentration and smaller pore size [71].

### 3.3. Mechanical Properties

Silkworm SF has a toughness of 80–78 MJ m-3 and exhibits an ultimate tensile strength (UTS) of 300–740 MPa. The breaking strain of this natural polymer is 4–26%, and the Young’s modulus of SF is 10–17 GPa. The presence of β-sheet crystallites determines the strength and stiffness of SF; thus, these structures impact the stability of this polymer [33]. Conversely, by removing the sericin, the UTS is impacted. In the biomaterial area, the mechanical strength and the elasticity of SF are remarkable properties. However, the properties of SF are impacted by several parameters, for instance the processing techniques and β-sheet patterns [72]. Nevertheless, it was reported that by influencing the molecular weight of SF, and its solvent composition, the stiffness of this polymer is impacted [73]. There are several studies that explored the impact of temperature on SF properties. By applying heat treatment on this natural polymer, its mechanical features changed, specifically the β-sheet patterns became predominant [32]. Conversely, numerous studies described complex strategies to improve the properties of SF by blending it with other polymers [74,75,76].

The extraordinary mechanical strength, elasticity and resistance of SF produced by *B. mori* are essential, especially for regenerative medicine and tissue regeneration applications [77]. Keeping this in mind, Chen et al. (2018) [78] developed 3D SF-based scaffolds in order to explore the potential of this biomaterial for tracheal defect repairing. They tested in vitro and in vivo the performance of SF scaffolds by analyzing the regeneration process of tracheal epithelium. Their data showed that SF scaffolds sustained tracheal mucosa regeneration within four weeks [78]. Another research group developed SF scaffolds by using high concentrations of SF aqueous solutions. Two different methods were combined for preparing the scaffolds, namely the salt-leaching and freeze-drying techniques. It was observed that the concentration of SF directly impacted the mechanical properties of the scaffolds. The authors revealed that after 30 days the biomaterials maintained their initial structure, suggesting that SF scaffolds are an appropriate choice for cartilage regeneration [79]. Table 1 points out the mechanical differences between several polymers.

### 3.4. Biologically Functional Properties

One of the most complex processes in the human body is wound healing. This process consists of four different phases, specifically hemostasis, inflammation, cell proliferation and resolution. However, there are numerous factors that affect this process [85]. In consequence, major efforts are being made for the development of biomaterials that promote wound healing, such as oxygenation, infections, or age and gender [86]. SF’s extraordinary properties are continually being studied for numerous applications in the medical area, including the process of wound healing. Mrowiec et al. (2012) [87] investigated the impact of fibroin and sericin in the wound healing process. Furthermore, they explored the molecular basis of biological functional properties of the two proteins. For this purpose, the authors used breast cancer (MDA-MB-231) and epithelial (Mv1Lu) cells and performed the wound healing scratch assessment. Their results confirmed that the silk proteins induced cell migration. Moreover, they determined that fibroin and sericin are involved in two signaling pathways, namely ERK and JNK, which are members of the family of mitogen-activated protein kinases. By phosphorylating the c-Jun, the two kinases activate and upregulate it [87].

Besides the fact that SF promotes cell migration, it has been shown that this protein improves cell adhesion. Nikam et al. (2020) [88] investigated the impact of SF nanofibers on fibroblast-like cells. After culturing the target cells on silk nanofibers for one week, their data showed that SF significantly improved cell adhesion [88].

Due to SF’s great characteristics, it has been shown that it is a promising biomaterial as a coating agent in terms of drug delivery [89]. In a recent study, SF was used by Kwon et al. (2021) [90] for coating probiotic strains. The aim of this study was to improve the stability of probiotics in the human body. By performing this experiment, the authors observed that the survival rate of the target probiotics that were SF coated was improved. Furthermore, by using SF as a biomaterial, cell adhesion was significantly increased [90].

### 3.5. Enhanced SF by Genetic Engineering

Aiming to meet the high demand of the medical field of biomaterials, numerous efforts have been made by the scientific community. For this purpose, in the last decades, genetic engineering has made extraordinary progress. In this direction, various studies have reported the development of enhanced SF fibers (Table 2) [91,92,93]. 

Spider silk exhibits superior mechanical properties that could be extraordinary in the biomaterials branch. It has been shown that the great toughness and elasticity of spider silk are determined by the presence of major ampullate silk protein (MaSp). The MaSp protein has a large and highly repetitive sequence, and due to this fact, it could not be obtained by using bacteria, yeast or plants [94]. Conversely, *B. mori* possesses the ability of spinning highly repetitive proteins. Xu et al. (2018) [24] obtained SF with enhanced mechanical properties by genetically manipulating the *B. mori* genome. The authors simultaneously knocked-out the *FibH* gene and knocked-in the major ampullate spidroin-1 gene from N. clavipes. On the same page, Kuwana et al. (2014) [94] obtained transgenic *B. mori* that expressed an altered dragline spider protein from *Araneus ventricosus*. By performing this, they obtained a tougher silk; furthermore, this mechanical property was improved by 53% [94].

**Table 2:** Advances in genetically manipulated *B. mori* for obtaining enhanced SF.

Exogenous Gene	Enhanced SF	Reference
Insulin-like Growth factor-1	Displays improved strength, elongation and tenacity	[95]
Human acidic fibroblast growth factor	Promotes cell proliferation	[96]
Human basic fibroblast growth factor and transforming growth factor-b1	Promotes cell proliferation and exhibits anti-inflammatory activity	[97]
Cecropin B and moricin	Antimicrobial activity	[98]
Green fluorescent protein and cecropin	Antibacterial activity and fluorescence	[99]
Enhanced green fluorescent protein, DsRed monomer fluorescent protein and monomeric Kusabira orange	Exhibits fluorescence	[100]
Laminin and fibronectin peptide adhesive fragments	Exhibits increased adhesive activity	[101]
Polyalanine motifs	Displays improved mechanical properties	[102]
Collagen and fibronectin	Improves cell adhesive properties	[103]

## 4. SF as a Green Material

**Figure 3:**
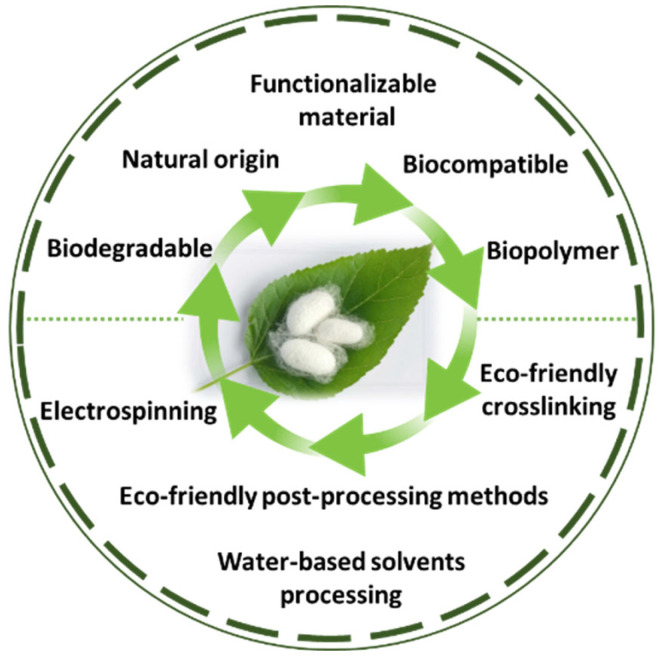
The main properties and methods of processing SF as a green material.

SF is a protein produced by the silkworm *B. mori*, and it is an extensively researched material with applications in various fields, including the cosmetics industry, biomedicine, and biomaterials [104,105]. Despite having a natural origin and being a biocompatible, mechanically superior, biodegradable and functionalizable material [33], the processing methods of SF oftentimes include highly complex processes that can have a damaging impact on the environment. This disadvantage comes in contradiction with one of the main motivators of researching SF’s potential as a biomaterial, which is its eco-friendly origin and properties (Figure 3). Moreover, the way that processing techniques can affect its microstructure and properties is still not fully understood. 

The classical regeneration of SF largely includes the use of toxic organic solvents, including calcium chloride/formic acid [106,107,108,109], hexafluoroisopropanol (HFIP) [110,111,112], lithium salt solutions such as lithium thiocyanate (LiSCN) [113] and lithium bromide (9.3 M LiBr-H_2_O) [114] and calcium nitrate/methanol (Ca(NO_3_)_2_)/CH_3_OH) mixtures [115]. Unfortunately, these solvents are hazardous and tend to be environmentally unfriendly [116]. Furthermore, they can directly affect the characteristics of SF and particularly impact the mechanical properties of the resulting silk biomaterials in subsequent processing [117,118].

Along with legislation and evolving attitudes toward environmental issues, new (ecological) alternative solvents that are used for SF processing have appeared. These include 0.02 M sodium carbonate (Na_2_CO_3_) [114], N-methyl morpholine-N-oxide (NMMO) [119], ionic liquids [120,121,122] and the so-called Ajisawa’s reagent consisting of calcium chloride/water/ethanol (CaCl_2_/H_2_O/C_2_H_5_OH) [123].

Water-based solvents have a lower environmental impact, and interest in their potential use in the processing of SF has gradually increased. Reizabal et al. (2021) [124] analyzed the effect of two dissolving techniques (CaCl_2_/H_2_O/EtOH ternary and LiBr/H_2_O binary solutions), three regeneration approaches (gas foaming, lyophilization, gelation), and one post-processing method (ethanol—EtOH) on porosity, macro- and microstructure, molecular and structural conformation, thermal behavior, water uptake and stability, and last but not least, heavy metal adsorption. Their results were promising, showing that it is possible to control SF properties through processing and that the green processing methods have the potential of expanding, even further, the applicability of SF-based biomaterials [124].

Bae et al. (2020) [125] prepared a water-soluble SF derivative by the methacrylation (introduction of glycidyl methacrylate—GMA) of SF’s reactive side chains (−NH_2_, −OH, −COOH). Through a green, eco-friendly electrospinning process using distilled water as a spinning solvent, GMA-modified SF water-soluble ultrathin fibers were obtained. Next, a structural change from random coils to b-sheets was facilitated by the treatment with an aqueous ethanol solution in order to insolubilize the fibers, and chemical crosslinking improved the water resistance. Electrospinning is a suitable alternative for the green processing of SF without the use of toxic substances [125]. Another study by Yang et al. (2017) [10] reported the feasibility of developing natural green composite matrices based on SF and MN (Manuka honey), used as an antimicrobial dressing in wound healing (ulcer). The SF was dissolved in a ternary solvent system of a CaCl_2_/H_2_O/CH_3_CH_2_OH solution (1:8:2 in molar ratio) and was mixed with various amounts of MH over 12 h to obtain stable spinning solutions. Subsequently, the mixtures loading with different amounts of MH were manufactured by a process of green electrospinning, where the ecological damage associated with solvents used is often overlooked [126]. In this regard, SF played a key role in indicating permeability of these membranes and in biocompatibility.

Freezing-induced silk I crystallization is another entirely green alternative for obtaining a water-insoluble SF material with the silk I crystalline structure from an SF aqueous solution. Crystallization induction allows for the obtainment of a water-insoluble silk I structure without any highly processing approaches or the use of organic solvents. Through a freezing-generated concentration and thermodynamically driven assembly, the silk I crystallization process can be controlled and can be environmentally friendly [127].

Yang et al. (2016) [128] improved the strain performance, up to 120%, of a green electrospun SF nanofiber through an eco-friendly 24 h process of hyaluronic acid (HA)/1-ethyl-3-(3-dimethylaminopropyl) carbodiimide (EDC)/N-hydroxysuccinimide (NHS)-crosslinking. Not only did their SF nanofibrous matrices have better tensile performance than the matrices treated conventionally (ethanol soaking or fumigating), but the hydrophilicity was higher, and the cytocompatibility was good. Thus, HA/EDC/NHS-crosslinking might be an eco-friendly option to improve the mechanical properties of SF nanofibers, which are the main restriction in their use as tissue engineering scaffolds [128].

Fei et al. (2013) [129] used SF as a biotemplate to produce silver nanoparticles in situ under light (both incandescent light and sunlight) at room temperature. The composite solution (RSF-AgNPs) was prepared by an ecological method where the AgNO_3_ powder was added to RSF solution and mixed until a transparent RSF-AgNO_3_ solution formed. The mixture solution obtained was then exposed under the light with an incandescent bulb and was incubated at room temperature for 24 h to produce an RSF-AgNPs composite solution. In this case, SF serves as the reducing agent of silver and the dispersing and stabilizing agent of the resulting silver nanoparticles. As the reaction does not need any other chemicals and only uses light as a power source, the synthetic route of silver nanoparticles reported here is environmentally friendly and energy saving. 

Raho et al. (2020) [130] used a green synthesis approach to create a composite hydrogel (CoHy) of carboxymethylcellulose-Na (CMC-Na) stabilized and loaded with AgNPs. An RSF solution was obtained by dissolving the fibroin with a solution of CaCl_2_ (CaCl_2_/CH_3_CH_2_OH/H_2_O) instead of LiBr and mixing it with a CMC-Na aqueous solution. The silver nitrate (AgNO_3_) was weighed and slowly added to the solution. For a great crosslinking, the composite solution was sonicated and exposed to UV radiation (for green synthesis of AgNPs). In this work, the synthesis of the hydrogel (CoHy) was based on a green process with ecological methods and natural components such as SF that served as a reducing agent in the green synthesis of AgNPs [129,131]. 

Switching toward eco-friendlier processing techniques of SF has gained popularity in materials science research and has raised the awareness of the negative impact of more environmentally toxic approaches. More research is necessary in order to find the ideal methods for maximizing SF’s potential as a biomaterial, with the lowest impact on the environment.

## 5. SF and Tissue Engineering

The purpose of tissue engineering is to combine cells with scaffold materials and growth factors in order to help the regeneration of or even replace damaged tissue and/or organs. The need for a biomaterial matrix that could aid in the development of viable and biologically active tissue, either in vivo or in vitro, has increased in the last few years [132]. It has been proven that SF stimulates the attachment and growth of human cells [133]; thus, it is not surprising that it became a Food and Drug Administration (FDA)-approved biomaterial that can successfully be used as a scaffold in tissue engineering due to its unique properties and complex structure [2].

In order for it to be successfully used in tissue engineering, SF has to undertake a dissolution process, with different morphologies of the regenerated SF observed according to the different solvent systems used [134]. SF has a tightly packed structure; thus, it is insoluble in most of the solvents used for the dissolution of polymers for biotechnology applications, including water. Obtaining an SF solution requires the use of near-saturated bromides or thiocyanates and dialysis, after which the solution is unstable and hard to store for a longer time [135]. 

Additionally, multiple techniques have been used to process SF, including chemical (surface modifications only, entire material modification, and sonochemical methods) and enzymatic approaches (surface/bulk material modifications) [135]. From gas plasma treatments [136] and UV irradiation to more complex systems where SF films were first aminated by ammonia, NH_3_, and then covalently sulfonated by sulfur dioxide [137], chemical modifications of SF are still of interest, despite their disadvantages. Biochemical enzymatic treatments of SF that have been studied include protease XIV and a-chymotrypsin treatment of SF for corneal regeneration [138], proteinase K for a porous network by a co-precipitation process with hydroxyapatite solution [139], and collagenase IA [140]. Furthermore, the enzymatic grafting of biomolecules such as lactoferrin [141] and chitosan was also described [142], and they are beginning to reveal their potential in tissue engineering. 

Among the multiple and complex applications of SF in tissue engineering, SF dressings are a viable and promising option for the improvement of wound healing strategies through tissue engineering techniques [14]. Through the use of SF in developing sponges [143], hydrogels [144,145], SF micro- and nanoparticles [146], nanofibrous matrices [147], scaffolds, and composite films [148], SF and its potential applications in tissue engineering have been intensely studied in the last few years. 

SF dressings can also improve the management of chronic wounds, as shown by Li et al. (2017) [149]. They prepared a functionalized SF dressing with topical bioactive insulin release by coaxial electrospraying of aqueous SF solution under mild processing conditions. This was based on the ability of insulin to stimulate cell migration and certain wound recovery methods [150]. The encapsulated insulin in the inner layer of the SF microparticles could thus be gradually released up to 28 days. The therapeutic effects of these microparticles loaded into an SF sponge (obtained by multilayer loading and freeze-drying method) was evaluated in vivo on dorsal full-thickness wounds of diabetic Sprague–Dawley rats. The degree of wound healing by measuring the area of wound closure showed the promotion of wound healing through accelerated wound closure and stimulation of vascularization and collagen deposition [149]. 

Moreover, processed SF after extraction of silk–sericin was also studied as a potential material for tissue engineering of the anterior cruciate ligament (ACL). A six-cord silk wire-rope matrix was designed that, based on ultimate tensile strength, linear stiffness, yield point, and percent elongation at break, had similar mechanical properties with the human ACL cells. This silk matrix supported both the attachment, as well as the expansion and differentiation, of adult human progenitor bone marrow stromal cells [132].

A highly porous 3D silk scaffold was designed by Wang et al. (2005) [151] and was combined with autologous adult mesenchymal stem cells (MSCs) in an attempt to perform in vitro cartilage tissue engineering. After 3 weeks of cell cultivation, RT-PCR analysis for cartilage-specific ECM gene markers and histological and immunohistochemical evaluations of cartilage-specific ECM components showed that the MSCs took the chondrogenic pathway with zonal architecture, including spatial cell arrangement and type II collagen distribution similar to native cartilage tissue [151]. Since cartilage has a limited self-repair capacity, and the current approaches for treating cartilage damage are not good enough for restoring the lost functionality, the potential of silk scaffolds in cartilage tissue engineering is of great importance and should be further evaluated in vivo. More recently, a 4D bioprinting system including SF was designed by Kim et al. (2020) [152]. Their system was based on digital light processing 3D bioprinting technology and photopolymerizable SF bioink. After obtaining a 4D-bioprinted silk hydrogel and using it to make trachea mimetic tissue, they successfully implanted it in the damaged trachea of an 8-week-old rabbit and proved the applicability of this new bioprinting system in vivo [152].

In another study, Mallepally et al. (2015) [153] used CO_2_-assisted acidification for the synthesis of SF hydrogels that were subsequently converted to SF aerogels using the salt-leaching method [154]. Moreover, these SF aerogels were structurally, mechanically and in vitro biologically characterized using human foreskin fibroblast cells. The results showed that the aqueous SF concentration plays an important role in tuning the morphology and textural properties of the SF aerogels. These presented a significantly higher surface area and pore volume compared to the freeze-dried SF scaffolds. Furthermore, the SF-aerogels presented are highly porous with an interconnected network of nanofibrils, contributing to the success of cytocompatibility with human foreskin fibroblasts cells and their propagation. The SF aerogel scaffolds obtained by supercritical CO_2_ have the potential for applications in tissue engineering, and the method of their synthesis can be used as an alternative method for 3D scaffold preparation.

Pacheco et al. (2020) [155] reported the use of several biopolymers in the development of SF/CS/SA multilayer membranes as a system for controlled drug release in wound healing. In this regard, the aim of the paper was to combine, in a single-composite material, the mechanical and biocompatibility properties of SF, the antimicrobial action of chitosan (CS) and the ideal exuded absorption of alginate (AS), thus achieving a synergistic effect. The membranes were prepared by pouring, where diclofenac sodium was incorporated as a model drug, into the chitosan solution before the solvent evaporated, which was stored in the middle layer of the membrane. The results showed that the incorporation of the drug did not affect the mechanical, thermal or barrier properties. Drug release was evaluated in vitro using a simulated solution of a wound exudate at 37 °C, where Fickian diffusion behavior was shown to be the dominant release mechanism. The results supported the idea that these multilayer membranes (SF/CS/SA multilayer membranes) could serve in biomedical applications as high-performance wound dressings.

**Figure 4:**
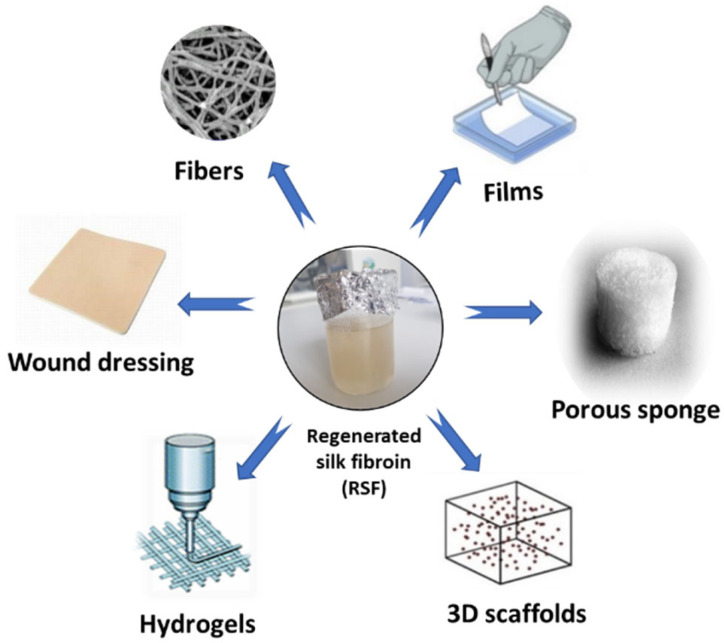
SF as a functional biomaterial for the tissue engineering field.

Among the varieties of materials tested in the field of tissue engineering, SF continues to be of increasing interest in the medical field, this being a promising material in scaffold (3D) fabrication, mimicking the natural extracellular matrix (Figure 4). Furthermore, the ease of processing, the excellent biocompatibility, the remarkable mechanical properties and the personalized degradability of SF make it a competitive material in tissue engineering and regenerative medicine.

## 6. SF Involved in the Treatment of the Eye, Bone and Skin Regeneration

### 6.1. SF Involved in the Treatment of the Eye

The cornea is a half-millimeter thick structure with three types of tissues: corneal epithelium (anterior to an acellular area—Bowmann’s layer), avascular corneal stroma with mesenchymal cell types (keratocyte), and an innermost single layer of corneal endothelial cells (posterior to an acellular zone—Descemet’s membrane). 

Harkin et al. (2011) [156] hypothesized that, among the complex eye structures that SF could be used as for a bioengineered tissue replacement would be the corneoscleral limbus, the corneal stroma, the corneal endothelium, and Ruysch’s complex (the outer blood-retinal barrier). For the corneoscleral limbus, they believe that a composite material such as a prosthetic basement membrane could be used to grow epithelium on, which would support the exchange of nutrients and regulatory substances that must be present for the maintenance of the stem cells. Overlying this prosthetic basement membrane, they proposed a 3D scaffold that is more biomimetic for the stromal cells [156]. Although, for the corneoscleral limbus, transplant from a deceased donor has been associated with a high rejection rate [157], this type of procedure seems to be highly successful for corneal stroma reparation. The risk of transmission of different diseases through a transplant makes it desirable that viable alternatives from bioengineered tissues should be available. In order for a biomaterial to contribute to corneal stroma regeneration, it should support the growth of an epithelial layer and provide a vehicle for the transplantation of keratocytes. However, they should also maintain a high level of transparency, unlike corneoscleral limbus reconstruction. 

Two of the methods currently used for obtaining SF films for corneal applications are the centrifugal casting technique and the dry casting method. Lee et al. (2016) [158] used both of these methods and compared the results. Their results showed that the centrifugal method offers more benefits, with less surface roughness of the films, increased growth of corneal fibroblasts (primary human corneal keratocytes) and better tensile strength and transparency [158]. Thus, the centrifugal casting technique is a promising method in tissue engineering of SF films for corneal regeneration. 

The potential of SF as a biomaterial for corneal epithelial cell sheet generation has further been evaluated by Liu et al. (2012) [159], who compared the biological cell behavior of human and rabbit primary and immortalized corneal epithelial cells on SF and denuded human amniotic membrane (AM). The cells adhered to and proliferated on both SF and AM, and expression of DNp63a, a progenitor cell marker [160], and keratin 3/12, a differentiation marker [161], existed on both cell culture types. Moreover, after being cultured at the air–liquid interface for 7 days, cells on the SF formed a stratified graft with a 2- to 3-cell layering with compact columnar cells on the basal layer and squamous cells present on the apical layers [159]. Since no cytotoxic response or inhibition of cell growth was observed in the SF cultures, their study strongly supports the high biocompatibility of SF biomaterial to corneal epithelial cells and their potential for corneal regeneration.

Lawrence et al. (2009) [162] designed 2-micromillimeter thick, surface-patterned silk films with 0.5–5.0-micromillimeter pores and demonstrated human and rabbit corneal fibroblast proliferation, alignment and corneal extracellular matrix expression in both 2D and 3D cultures on these films. The thickness was carefully chosen to imitate corneal collagen lamellae dimensions, whereas the surface pattern was meant to guide cell alignment. Pores were used for the enhancement of translamellar diffusion of nutrients and promotion of cell–cell interactions [162]. Their results further strengthen the potential of SF in bioengineered tissues for corneal injuries and pathologies. 

The eye is an extremely complex organ, and its functionality in normal parameters highly influences the quality of life. Multiple research branches are currently performing joint work with the sole purpose of improving the regeneration of eye components. In this context, SF has proven to be a suitable candidate for a biomaterial that can be used for tissue engineering of eye components, especially for the cornea. Despite the enthusiasm that SF has seeded, more research is needed in order to better understand the interaction of its properties as a biomaterial and the human living organism, especially within the eye.

### 6.2. SF Used in the Treatment of the Bone

Bone is a rigid tissue with the essential propriety of providing structural support and protection of certain internal organs while supporting muscular contraction and serving as a deposit for certain minerals [163]. While it heals and remodels without leaving gross scars, efficient therapeutic strategies are needed for the efficient management of bone injuries. Bone grafts were the main option for a while, but their multiple disadvantages [164,165] have encouraged further research for viable alternatives. 

For this purpose, in a recent study, Zhao et al. (2022) [166] developed SF-based scaffolds. One of the key players when it comes to bone repair and healing processes is hydroxyapatite. Being the foremost mineral element in bones, the authors combined SF with hydroxyapatite and obtained porous scaffolds. Moreover, they developed poly(lactic-co-glycolic acid) (PLGA)-based microspheres for controlled release of naringin, which is known for its ability to promote stem cell differentiation into osteoblasts. The microspheres were attached to the developed scaffold. First, the authors used human bone mesenchymal stem cell cultures in order to investigate the impact of the developed biomaterial on stem cell differentiation potential. Their data showed that the microspheres inlaid into the developed scaffolds have great potential in promoting osteogenic differentiation of target cells. Second, they used rabbits as a model organism to evaluate the bone repair process; more specifically, they surgically induced significant lesions of distal femoral epiphysis. The results indicated that the obtained biomaterial has great potential in the bone regeneration area by promoting bone defect healing [166].

A study conducted by Meinel et al. (2005) [167] used SF to form porous scaffolds on which human mesenchymal stem cells (hMSC) were cultured for 5 weeks. Afterward, they used the tissue as an implant for mouse critical calvarial defects. After another 5 weeks, multiple analyses including gene expression analysis, biochemical assays, and X-ray diffractometry demonstrated that SF successfully induced advanced bone formation [167].

The properties of SF of providing an osteogenic environment for bone-related outcomes was also proven by Zhao et al. (2009) [168]. They used apatite-coated SF scaffolds seeded with osteogenically induced autologous bone marrow stromal cells (bMSCs). The graft was used to repair inferior mandibular border defects in a canine model in comparison with bMSCs and SF scaffolds (no apatite-coating), apatite-coated SF scaffolds alone, SF scaffolds alone, autologous mandibular grafts, and untreated blank defects. Four weeks after the operation, new bone formation was detected through histological and radiographic examinations, with the defects being completely repaired for the bMSCs together with the apatite-coated SF group [168]. Their favorable results suggest that SF scaffolds could successfully be used with an apatite coating in combination with osteogenically induced autologous bone marrow stromal cells for the management of bone defects through tissue engineering. 

Hydroxyapatite-coated SF composites were proven through fluorescence microscopy and scanning electron microscopy (SEM) to sustain cell adhesion and proliferation of mesenchymal cells derived from EGFP-expressing transgenic rat bone marrow in 10-day cultures. The results were comparable to the adhesion and growth of the cells seeded on tissue culture polystyrene dishes, which are standard scaffolds for the culture of cells, and which strongly support the use of SF scaffolds for regenerative medicine with applications in bone-related pathologies [169]. 

Another novel nanocomposite containing SF that might be used as tissue engineering scaffold or bone replacement was obtained by Cui et al. (2007) [170]. During its synthesis in an aqueous solution containing SF, hydroxyapatite (HA) nanocrystallites nucleated and grew preferentially along the plane (002) of the HA crystal, suggesting that SF might have an essential role in the mineralization of HA nanocrystallites. Moreover, the resulting nanocomposite had a compression strength of 97.6 MPa, which is higher than that of woven bone [170]. This compression strength was much higher than the one found in the case of the SF-chitosan/nano-hydroxyapatite porous scaffolds by Wen et al. (2007) [171]. Although their scaffold had good bioactivity, proven in the simulated body fluid experiments, when a layer of bone-like apatite crystals that were randomly oriented formed on the scaffold surface, their compressive strength varied only from 0.26 to 1.96 MPa. Both the porosity and the compressive strength seemed to change in relation to organic phase concentration and pre-freezing temperatures, and the authors concluded that a pre-freezing temperature of −80 °C and 5% organic concentration would be the best choices for bone tissue engineering [171].

The multiple properties and functions that bones have in the body make it necessary to have more than one viable option for bone regeneration, especially in the context of the high frequency of accidents involving bony structures. Considering the disadvantages of bone grafts and the impact that post-traumatic bone lesions can have on the quality of life through the impairment it causes, SF research as a potential tissue engineering biomaterial for bone-replacement structure synthesis has recently gained popularity. Although this potential still has to be tested in vivo, the properties of SF in current research, as well as the in vitro experimental work that has been performed, suggest that this is the proper direction to go.

### 6.3. SF Involved in the Treatment of Skin Regeneration

Skin has a complex function, including protection, thermo-regulation and a barrier between the outside of the body and the underlying tissues. Because skin lesions are frequent, over time, multiple approaches have been suggested for the proper treatment of injuries and pathologies developed at this level. Skin grafts have multiple classifications (based on thickness, donor origin, etc.), and each has different advantages and disadvantages. Their limitations of size, donor site availability and tolerability make skin grafting an imperfect option, and they have encouraged the search for a bioengineered skin equivalent [172].

A complex SF-based formic acid-crosslinked 3D nonwoven scaffold was studied by Dal Pra et al. (2006) [173] and was proven to be a great candidate for human tissue engineering. Among the methods they used was scanning electron microscopy (SEM) and differential scanning calorimetry, as well as thermogravimetric and tensile strength studies. These showed that the scaffolds are a composite material with anisotropic SF fibers enclosed within an isotropic matrix of SF in film form. They also obtained fibers, which as well as films, were firmly crosslinked and water insoluble. The crosslinking was hypothesized to have been induced by formic acid treatment and the lack of water solubility to their ß-sheet crystalline structure. Both human embryonic kidney cells and human dermal fibroblasts were attached to the scaffolds within 3 h and started proliferating and colonizing the scaffolds after 24 h. The cultures kept growing, with an active metabolism and no sign of protein catabolism, for at least 15 weeks [173].

A chitosan/SF blend film was evaluated as a biomaterial for skin tissue engineering by Luangbudnark et al. (2012) [174]. Chitosan appears to have enhanced β-sheet conformation of fibroin as well as increased flexibility, enzyme degradation, and swelling index. Fourier transform infrared spectroscopy and differential scanning calorimetry analysis showed intermolecular interactions between chitosan and SF. Furthermore, XTT assay showed no toxicity of the chitosan/SF blend film on fibroblast cell cultures, which showed proliferation. The blend films had a percentage of elongation at the break between 6.4% and 14.4%, which seemed to increase proportionally with the chitosan content, while the tensile strength was between 52.8 and 58.3 MPa, independent of the chitosan proportion. Conversely, increasing the fibroin content of the CS/SF blend films led to lower flexibility [174].

Another type of recently studied blended scaffold of SF was in combination with human hair keratin with a potential use as a dermal substitute [175]. Three-dimensional (3D) blended scaffolds were fabricated by freeze-drying and were tested on the L929 mouse-fibroblastic cell line. Functional fibroblasts showed good biocompatibility and proliferation on the 3D scaffolds, with a greater expression of collagen type I proven by immunohistochemical staining methods. Cellular compatibility evaluated through MTT assay showed good cell viability for 14 days of culture. Further, these composite scaffolds supported cell attachment and proliferation along with intact extracellular matrix (ECM) deposition, specifically for Col I. These results demonstrate that SF–keratin blended scaffolds are promising substrates for dermal reconstruction, wound healing, and other biomedical applications [175]. 

**Figure 5:**
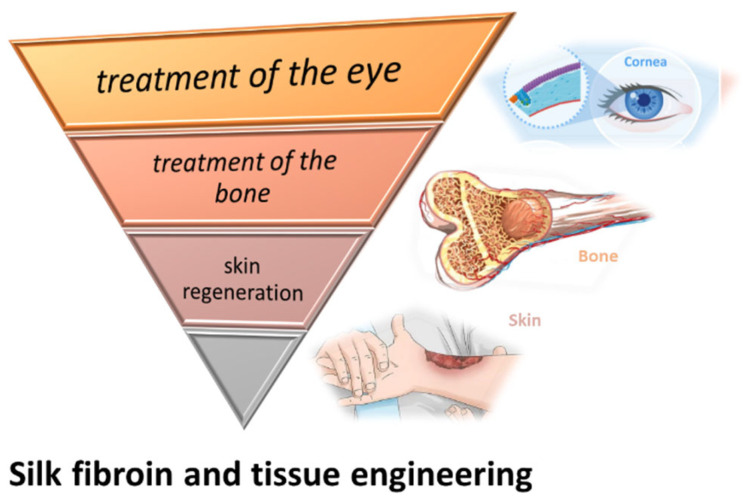
SF involved in the treatment of the eye, bone, and skin regeneration.

Although, for the treatment of skin injuries, skin grafting has been studied and used for a long time, they have a limitation of size, are dependent on donor size availability, and have the potential to be rejected by the host organism. These limitations cannot be overlooked, especially in complex and extended burn-cases that require additional support and special care. The biocompatibility of SF, as well as its natural origin and abundance, give it the potential to overcome certain issues of skin grafts (Figure 5). Besides its potential use for bioactive wound dressings, SF might become an important resource for in vivo regeneration of the skin.

## 7. SF in Cancer Therapy

Globally, cancer is the leading cause of mortality [176]. Currently, chemotherapy is the standard therapy for numerous types of cancer. However, there are numerous disadvantages when it comes to chemotherapy, the most important being its high toxicity [177]. Keeping these aspects in mind, there is a major need for the development of drugs that exhibit reduced toxicity. Due to its extraordinary properties, SF has great potential as a drug delivery system. Another great advantage of using SF is its great feasibility of modification and the ease of processing [178,179]. Being extremely versatile, SF has been used to deliver numerous compounds, such as vaccines, proteins, therapeutic compounds and so on. Sun et al. (2019) [180] encapsulated doxorubicin in SF-based nanoparticles for exploring its feasibility in target cancer therapy. In addition, on the nanoparticles’ surface, the authors covalently grafted folic acid in order to target the tumor cells. Their results showed that the prepared SF nanoparticles did not affect cell viability or proliferation. This approach displayed promising results, as the particles were able to target the tumor cells. Moreover, the doxorubicin was controllably released [180]. 

In a recent study, Saqr et al. (2021) [181] developed SF nanoparticles that were loaded with docetaxel to target breast cancer cells. Their data revealed that the developed nanoparticles exhibited an enhanced cytotoxic effect against the targeted cancer cells [181]. Mottaghitalab et al. (2017) [182] encapsulated gemcitabine in SF nanoparticles in order to target lung cancer cells. As an experimental model animal, the authors used mice. The results of this approach showed that SF nanoparticles were able to reduce the side effect of gemcitabine. Furthermore, the therapeutic activity resulting from this formulation was higher than other approaches [182]. In the same year, Moin et al. (2021) [183] developed both nano- and micro-fibroin-based particles as drug delivery systems to encapsulate doxorubicin. The authors used two types of cancer cell lines, specifically MCF-7 and SAOS2, and one normal human cell line, namely HFF. By using the MTT assessment, they evaluated the toxicity exhibited by micro- and nanoparticles on cell lines. Their data showed that cell growth was inhibited. Furthermore, the impact of SF-based particles on *p53* expression was evaluated. Numerous studies have highlighted that *p53* overexpression plays a key role when administering doxorubicin to cancer patients; specifically, the overexpression if this gene is linked to higher sensitivity of malignant cell sensitivity to this chemotherapy agent. By using the real-time PCR method to evaluate *p53* expression, the doxorubicin-loaded SF nanoparticles induced higher *p53* expression in the malignant cell lines and decreased its expression in the normal cell line. Conversely, the doxorubicin-loaded SF microparticles decreased *p53* expression in the SAOS cell line and significantly increased it in the MCF-7 and HFF cell lines. Moreover, the authors investigated the reactive oxygen species levels after treating the cell lines with the developed biomaterials and observed that the reactive oxygen species levels were lower in two cell lines, namely MCF-7 and HFF. These findings highlight the great contribution of SF-based biomaterials in cancer therapy [183]. 

In 2022, a group of researchers successfully developed SF-based double-layer microneedles. The authors developed the delivery devices in order to encapsulate and administer triptorelin in a controlled released manner. Triptorelin is widely used in the treatment of prostate gland carcinomas due to its inhibitory effect on testosterone; however, it is an injectable suspension that exhibits certain disadvantages such as cold-chain storage or the administration method. After developing the SF-based microneedles and successfully encapsulating triptorelin in the microneedles’ tip, the authors subcutaneously implanted the devices in rats in order to evaluate its therapeutic effects. The authors showed that by encapsulating triptorelin into SF microneedles, its half-life was extended compared with the half-time obtained after directly subcutaneously injecting the drug. In order to confirm the inhibitory effect of encapsulated triptorelin on testosterone concentration, as controls, castrated and healthy rats were used. Their results showed that by using the developed microneedles to administer triptorelin, the testosterone levels were the same as the levels observed in the castrated rats, and the inhibitory effect was maintained for more than seven days. Conversely, by subcutaneously injecting the target drug, the repressive action of triptorelin was maintained for less than one day. These findings suggest that SF-based microneedles are a promising approach for delivering triptorelin [184].

**Figure 6:**
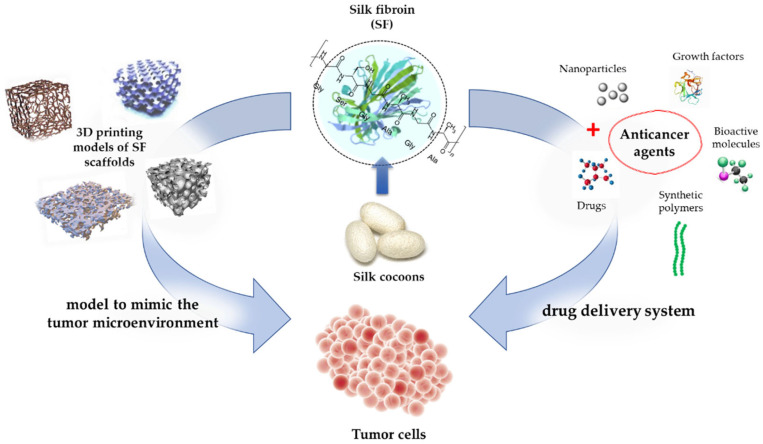
SF-based drug delivery and the production of 3D SF scaffolds for the growth of cancer cells.

Furthermore, SF is currently being used as a tumor model to investigate cancer biology. By using a tumor model to mimic the tumor microenvironment, valuable insights could be observed. Dondajewska et al. (2018) [185] used an SF-based scaffold to construct a breast cancer model. The model provided insights into the interactions between the stromal fibroblasts and cancer cells. This complex model represents a promising approach for expanding the understanding of tumor biology [185]. In another study, scaffolds were developed that contained both SF and chitosan to mimic a tumor’s microenvironment. These scaffolds allow for the investigation of tumor drug resistance mechanisms and provide insights for the evaluation of therapeutic agents [186]. Table 3 shows numerous studies that successfully used SF in cancer therapy, and Figure 6 illustrates the use of SF in cancer therapy.

## 8. Conclusions

SF represents the major structural element of the silk produced by *B. mori*. Numerous studies have demonstrated the unique properties of this protein. One of the most important characteristics is the great level of biocompatibility with human organisms. Moreover, SF has great mechanical properties and biodegradable behavior. Due to its unique properties, this protein has an extraordinary potential in the medical field and beyond. It has been used for a long time as a suture in surgeries, but currently, it is the main component of numerous biomaterials. Great advances have been made in the medical area due to SF’s ability to generate numerous biomaterials, such as scaffolds or nanoparticles. Moreover, by applying different techniques, SF’s features can be controlled and remodeled. In recent years, SF has gained attention due to the fact that it can be considered a green material.

SF is currently being used in various areas, and a wide range of techniques has been employed for obtaining different types of SF-based biomaterials. There have been numerous types of scaffolds obtained by using this natural polymer, such as hydrogels, films, nanospheres, sponges and coatings. With the current progress that is being made for a better understanding of SF’s structure, properties, and processing methods, there are new opportunities for its applicability. Moreover, SF has been associated with a wide range of compounds in order to maximize its mechanical and biological functional properties. There have been various studies that have used SF for the cornea, bone, and its applications. Beside SF’s extraordinary contribution to tissue engineering, this biomaterial has a great input in cancer therapy. It is currently being used as a coating agent in the treatment of several types of cancers, for instance in lung and breast cancer. Furthermore, molecular engineering has been applied in order to obtain enhanced SF for biomedical applications.

## Figures and Tables

**Table 1:** Comparison between the mechanical properties of several polymers.

Polymer	Source	UTS (MPa)	Modulus (GPa)	Breaking Strain (%)	References
SF	*B. mori*	300–740	10–17	4–26	[33]
Silk	*B. mori*	740	10	20	[80]
Silk	N. clavipes	875	10.9	16.7	[33]
Polylactide	Corn	69.8 ± 3.2	1777 ± 42	5.7 ± 0.3	[81]
Polyethylene-terephthalate	Synthetic	56	2.2	7300	[82]
Polypropylene	Synthetic	34.5	1.7	400	[82]
Cellulose	Bacteria	11.6 ± 0.8	180.3 ± 10.6	8.2 ± 0.6	[83,84]

**Table 3:** SF-based biomaterials used in cancer therapy.

Biomaterial	Type of Cancer	Reference
SF hydrogels	Hepatocellular carcinoma	[187]
SF–Thelebolan matrix	Soft tissue carcinoma	[188]
Doxorubicin loaded SF nanoparticles	Brain cancer	[189]
SF–Sodium alginate nanocarriers	Colorectal cancer	[190]
SF-based metastasis model	Breast cancer	[191]
Triptolide–Celastrol-loaded SF nanoparticles	Pancreatic cancer	[192]
Alpha-mangostin loaded SF nanoparticles	Colon cancer; Breast cancer	[193]
SF rods	Breast cancer	[194]
Quercetin loaded SF nanoparticles	Breast cancer; Lung metastasis	[195]
Biliverdin–SF hydrogel	Glioma	[196]
Floxuridine-loaded SF nanospheres	Digestive tract cancer; Lung cancer	[197]
Curcumin-loaded SF nanoparticles	Breast cancer	[198]

## Data Availability

Not applicable.

## References

[1] Ozkale B., Selman M., Mooney D.J. (2021). Active Biomaterials for Mechanobiology. Biomaterials.

[2] Sun W., Gregory D.A., Tomeh M.A., Zhao X. (2021). Silk Fibroin as a Functional Biomaterial for Tissue Engineering. Int. J. Mol. Sci..

[3] Holland C., Numata K., Rnjak-Kovacina J., Seib F.P. (2019). The Biomedical Use of Silk: Past, Present, Future. Adv. Healthc. Mater..

[4] Sunija A.J. (2018). Biomaterials and Biotechnological Schemes Utilizing TiO_2_ Nanotube Arrays—A Review. Fundamental Biomaterials: Metals.

[5] Shanmugam K., Sahadevan R. (2018). Bioceramics—An Introductory Overview.

[6] Sionkowska A. (2011). Current Research on the Blends of Natural and Synthetic Polymers as New Biomaterials: Review. Prog. Polym. Sci..

[7] Ullah S., Chen X. (2020). Fabrication, Applications and Challenges of Natural Biomaterials in Tissue Engineering. Appl. Mater. Today.

[8] Wu R., Li H., Yang Y., Zheng Q., Li S., Chen Y. (2021). Bioactive Silk Fibroin-Based Hybrid Biomaterials for Musculoskeletal Engineering: Recent Progress and Perspectives. ACS Appl. Bio Mater..

[9] Kostag M., Jedvert K., El O.A. (2021). Engineering of Sustainable Biomaterial Composites from Cellulose and Silk Fibroin: Fundamentals and Applications. Int. J. Biol. Macromol..

[10] Yang X., Fan L., Ma L., Wang Y., Lin S., Yu F., Pan X., Luo G., Zhang D., Wang H. (2017). Green Electrospun Manuka Honey/Silk Fibroin Fibrous Matrices as Potential Wound Dressing. Mater. Des..

[11] Tao G., Cai R., Wang Y., Liu L., Zuo H., Zhao P., Umar A., Mao C., Xia Q., He H. (2019). Bioinspired Design of AgNPs Embedded Silk Sericin-Based Sponges for Efficiently Combating Bacteria and Promoting Wound Healing. Mater. Des..

[12] Bakhsheshi-Rad H.R., Fauzi A., Aziz M., Akbari M., Hadisi Z., Omidi M., Chen X. (2020). Development of the PVA/CS Nano Fibers Containing Silk Protein Sericin as a Wound Dressing: In Vitro and in Vivo Assessment. Int. J. Biol. Macromol..

[13] Zhou L., Yu K., Lu F., Lan G., Dai F., Shang S., Hu E. (2020). Minimizing Antibiotic Dosage through in Situ Formation of Gold Nanoparticles across Antibacterial Wound Dressings: A Facile Approach Using Silk Fabric as the Base Substrate. J. Clean. Prod..

[14] Patil P.P., Reagan M.R., Bohara R.A. (2020). Silk Fibroin and Silk-Based Biomaterial Derivatives for Ideal Wound Dressings. Int. J. Biol. Macromol..

[15] Zakeri-Siavashani A., Chamanara M., Nassireslami E., Shiri M., Hoseini-Ahmadabadi M., Paknejad B. (2022). Three Dimensional Spongy Fibroin Scaffolds Containing Keratin/Vanillin Particles as an Antibacterial Skin Tissue Engineering Scaffold. Int. J. Polym. Mater. Polym. Biomater..

[16] DeBari M.L., King C.I., Altgold T.A., Abbott R.D. (2021). Silk Fibroin as a Green Material. ACS Biomater. Sci. Eng..

[17] Benfenati V., Toffanin S., Chieco C., Sagnella A., Virgilio N. Di, Posati T., Varchi G., Natali M., Ruani G., Muccini M. (2019). Silk Fibroin Based Technology for Industrial Biomanufacturing. Factories of the Future.

[18] Hu W., Lu W., Wei L., Zhang Y., Xia Q. (2019). Molecular Nature of Dominant Naked Pupa Mutation Reveals Novel Insights into Silk Production in *Bombyx mori*. Insect Biochem. Mol. Biol..

[19] Song J., Chen Z., Liu Z., Yi Y., Tsigkou O., Li J., Li Y. (2021). Controllable Release of Vascular Endothelial Growth Factor (VEGF) Bywheel Spinning Alginate/Silk Fibroin Fibers for Wound Healing. Mater. Des..

[20] Pérez-Rigueiro J., Ruiz V., Cenis J.L., Elices M., Pugno N.M. (2020). Lessons From Spider and Silkworm Silk Guts. Front. Mater..

[21] Xu J., Dong Q., Yu Y., Niu B., Ji D., Li M., Huang Y., Chen X. (2018). Mass Spider Silk Production through Targeted Gene Replacement in *Bombyx mori*. Proc. Natl. Acad. Sci. USA.

[22] Etebari K., Mirhoseini S.Z., Matindoost L. (2005). A Study on Interaspecific Biodiversity of Eight Groups of Silkworm (*Bombyx mori*) by Biochemical Markers. Insect Sci..

[23] Abdelli N., Peng L., Keping C. (2018). Silkworm, *Bombyx mori*, as an Alternative Model Organism in Toxicological Research. Environ. Sci. Pollut. Res..

[24] Xu H., O’Brochta D.A. (2015). Advanced technologies for genetically manipulating the silkworm Bombyx mori, a model lepidopteran insect. Proc. R. Soc. B Biol. Sci..

[25] Zhu Z., Imada T., Asakura T. (2009). Preparation and Characterization of Regenerated Fiber from the Aqueous Solution of *Bombyx mori* Cocoon Silk Fibroin. Mater. Chem. Phys..

[26] Marín C.B., Fitzpatrick V., Kaplan D.L., Landoulsi J., Guénin E., Egles C. (2020). Silk Polymers and Nanoparticles: A Powerful Combination for the Design of Versatile Biomaterials. Front. Chem..

[27] Wang F., Guo C., Yang Q., Li C., Zhao P., Xia Q., Kaplan D.L. (2021). Protein Composites from Silkworm Cocoons as Versatile Biomaterials. Acta Biomater..

[28] Qi Y., Wang H., Wei K., Yang Y., Zheng R.Y., Kim I.S., Zhang K.Q. (2017). A Review of Structure Construction of Silk Fibroin Biomaterials from Single Structures to Multi-Level Structures. Int. J. Mol. Sci..

[29] Wang J., Yan S., Lu C., Bai L. (2009). Biosynthesis and Characterization of Typical Fibroin Crystalline Polypeptides of Silkworm *Bombyx mori*. Mater. Sci. Eng. C.

[30] Ma Y., Luo Q., Ou Y., Tang Y., Zeng W., Wang H., Hu J. (2021). New Insights into the Proteins Interacting with the Promoters of Silkworm Fibroin Genes. Sci. Rep..

[31] Lotz B., Cesari F.C. (1979). The Chemical Structure and the Crystalline Structures of *Bombyx mori* Silk Fibroin. Biochimie.

[32] Vidya M., Rajagopal S. (2021). Silk Fibroin: A Promising Tool for Wound Healing and Skin Regeneration. Int. J. Polym. Sci..

[33] Koh L.D., Cheng Y., Teng C.P., Khin Y.W., Loh X.J., Tee S.Y., Low M., Ye E., Yu H.D., Zhang Y.W. (2015). Structures, Mechanical Properties and Applications of Silk Fibroin Materials. Prog. Polym. Sci..

[34] Dong Z., Zhao P., Zhang Y., Song Q., Zhang X., Guo P. (2016). Analysis of Proteome Dynamics inside the Silk Gland Lumen of *Bombyx mori*. Sci. Rep..

[35] Leem J.W., Fraser M.J., Kim Y.L. (2020). Transgenic and Diet-Enhanced Silk Production for Reinforced Biomaterials: A Metamaterial Perspective. Annu. Rev. Biomed. Eng..

[36] Panda D., Konar S., Bajpai S.K., Arockiarajan A. (2018). Thermodynamically-Consistent Constitutive Modeling of Aligned Silk Fibroin Sponges: Theory and Application to Uniaxial Compression. Int. J. Solids Struct..

[37] Michelle G., Agostini M., Moraes D., Cecília A., Rodas D., Zazuco O., Masumi M. (2011). Hydrogels from Silk Fibroin Metastable Solution: Formation and Characterization from a Biomaterial Perspective. Mater. Sci. Eng. C.

[38] Bassani G.A., Vincoli V., Biagiotti M., Valsecchi E., Zucca M.V., Clavelli C., Alessandrino A., Freddi G. (2022). A Route to Translate a Silk-Based Medical Device from Lab to Clinic: The Silk Biomaterials Srl Experience. Insects.

[39] Ghalei S., Handa H. (2022). A Review on Antibacterial Silk Fibroin-Based Biomaterials: Current State and Prospects. Mater. Today Chem..

[40] Khademolqorani S., Tavanai H., Chronakis I.S., Boisen A. (2021). The Determinant Role of Fabrication Technique in Final Characteristics of Scaffolds for Tissue Engineering Applications: A Focus on Silk Fibroin-Based Scaffolds. Mater. Sci. Eng. C.

[41] Jo Y., Kweon H., Kim D., Baek K., Chae W., Kang Y., Oh J., Kim S., Garagiola U. (2021). Silk Sericin Application Increases Bone Morphogenic Protein-2/4 Expression via a Toll-like Receptor-Mediated Pathway. Int. J. Biol. Macromol..

[42] Teuschl A., Griensven M. Van, Redl H., Teuschl A.H., Bioreactors E. (2014). Sericin Removal from Raw *Bombyx mori* Silk Scaffolds of High Hierarchical Order. Tissue Eng. Part C Methods.

[43] Gholipourmalekabadi M., Sapruc S., Samadikuchaksaraei A., Reis R.L., Kaplan D.L., Subhas C.K. (2019). Silk Fibroin for Skin Injury Repair: Where Do Things Stand?. Adv. Drug Deliv. Rev..

[44] Crakes K.R., Herrera C., Morgan J.L., Olstad K., Hessell A.J., Ziprin P., Liwang P.J., Dandekar S. (2020). Efficacy of Silk Fibroin Biomaterial Vehicle for in Vivo Mucosal Delivery of Griffithsin and Protection against HIV and SHIV Infection Ex Vivo. J. Int. AIDS Soc..

[45] Madden P.W., Klyubin I., Ahearne M.J. (2020). Silk Fibroin Safety in the Eye: A Review That Highlights a Concern. BMJ Open Ophtalmol..

[46] Yang Y., Chen X., Ding F., Zhang P., Liu J., Gu X. (2007). Biocompatibility Evaluation of Silk Fibroin with Peripheral Nerve Tissues and Cells in Vitro. Biomaterials.

[47] Zhang J., Huang H., Ju R., Chen K., Li S., Wang W., Yan Y. (2017). In Vivo Biocompatibility and Hemocompatibility of a Polytetra Fl Uoroethylene Small Diameter Vascular Graft Modified with Sulfonated Silk Fibroin. Am. J. Surg..

[48] Tian Y., Wu Q., Li F., Zhou Y., Huang D., Xie R., Wang X., Zheng Z., Li G. (2021). A Flexible and Biocompatible *Bombyx mori* Silk Fibroin/Wool Keratin Composite Scaffold with Interconnective Porous Structure. Colloids Surf. B Biointerfaces.

[49] Wang D., Wang L., Lou Z., Zheng Y., Wang K., Zhao L., Han W., Jiang K., Shen G. (2020). Biomimetic, Biocompatible and Robust Silk Fibroin-MXene Film with STable 3D Cross-Link Structure for Flexible Pressure Sensors. Nano Energy.

[50] Wang S., Zhang Y., Wang H., Dong Z. (2011). Preparation, Characterization and Biocompatibility of Electrospinning Heparin-Modified Silk Fibroin Nanofibers. Int. J. Biol. Macromol..

[51] Choi Y., Cho D., Lee H. (2022). Development of Silk Fibroin Scaffolds by Using Indirect 3D-Bioprinting Technology. Micromachines.

[52] Stoica A.E., Chircov C., Grumezescu A.M. (2020). Hydrogel Dressings for the Treatment of Burn Wounds: An Up-To-Date Overview. Materials.

[53] Zheng L., Li S., Luo J., Wang X. (2020). Latest Advances on Bacterial Cellulose-Based Antibacterial Materials as Wound Dressings. Front. Bioeng. Biotechnol..

[54] Yin C., Han X., Lu Q., Qi X., Guo C., Wu X. (2022). Rhein Incorporated Silk Fibroin Hydrogels with Antibacterial and Anti-Inflammatory Efficacy to Promote Healing of Bacteria-Infected Burn Wounds. Int. J. Biol. Macromol..

[55] Mu Y., Gage F.H. (2011). Adult Hippocampal Neurogenesis and Its Role in Alzheimer’s Disease. Mol. Neurodegener..

[56] Zhong S., Wang M., Zhan Y., Zhang J., Yang X., Fu S., Bi D., Gao F. (2020). Single-Nucleus RNA Sequencing Reveals Transcriptional Changes of Hippocampal Neurons in APP23 Mouse Model of Alzheimer’s Disease. Biosci. Biotechnol. Biochem..

[57] Tang X., Ding F., Yang Y., Hu N., Wu H., Gu X. (2008). Evaluation on in Vitro Biocompatibility of Silk Fibroin-Based Biomaterials with Primarily Cultured Hippocampal Neurons. J. Biomed. Mater. Res. A.

[58] Reinke J., Sorg H. (2012). Wound Repair and Regeneration. Eur. Surg. Res..

[59] Zhang W., Chen L., Chen J., Wang L., Gui X., Ran J. (2017). Silk Fibroin Biomaterial Shows Safe and Effective Wound Healing in Animal Models and a Randomized Controlled Clinical Trial. Adv. Healthc. Mater..

[60] Acharya C., Ghosh S.K., Kundu S.C. (2008). Silk Fibroin Protein from Mulberry and Non-Mulberry Silkworms: Cytotoxicity, Biocompatibility and Kinetics of L929 Murine Fibroblast Adhesion. J. Mater. Sci. Mater. Med..

[61] Kim S.H., Yeon Y.K., Lee J.M., Chao J.R., Lee Y.J., Seo Y.B., Sultan T., Lee O.J., Lee J.S., Yoon S. (2018). Precisely Printable and Biocompatible Silk Fibroin Bioink for Digital Light Processing 3D Printing. Nat. Commun..

[62] Cao Y., Wang B. (2009). Biodegradation of Silk Biomaterials. Int. J. Mol. Sci..

[63] Zhang L., Liu X., Li G., Wang P., Yang Y. (2019). Tailoring Degradation Rates of Silk Fibroin Scaffolds for Tissue Engineering. J. Biomed. Mater..

[64] Wang H., Zhang Y., Wei Z. (2021). Characterization of Undegraded and Degraded Silk Fibroin and Its Significant Impact on the Properties of the Resulting Silk Biomaterials. Int. J. Biol. Macromol..

[65] Wang Y., Fan S., Li Y., Niu C., Li X., Guo Y., Zhang J., Shi J., Wang X. (2020). Silk Fibroin/Sodium Alginate Composite Porous Materials with Controllable Degradation. Int. J. Biol. Macromol..

[66] Luo Z., Zhang Q., Shi M., Zhang Y., Tao W., Li M. (2015). Effect of Pore Size on the Biodegradation Rate of Silk Fibroin Scaffolds. Adv. Mater. Sci. Eng..

[67] Sun M., Li Q., Yu H., Cheng J., Wu N., Shi W., Zhao F., Shao Z., Meng Q., Chen H. (2022). Cryo-Self-Assembled Silk Fibroin Sponge as a Biodegradable Platform for Enzyme-Responsive Delivery of Exosomes. Bioact. Mater..

[68] Catto V., Farè S., Cattaneo I., Figliuzzi M., Alessandrino A., Freddi G., Remuzzi A., Cristina M. (2015). Small Diameter Electrospun Silk Fibroin Vascular Grafts: Mechanical Properties, in Vitro Biodegradability, and in Vivo Biocompatibility. Mater. Sci. Eng. C.

[69] Mehrabani M.G., Karimian R., Mehramouz B., Rahimi M., Kafil H.S. (2018). Preparation of Biocompatible and Biodegradable Silk Fibroin/Chitin/Silver Nanoparticles 3D Scaffolds as a Bandage for Antimicrobial Wound Dressing. Biol. Macromol..

[70] Fan H., Liu H., Toh S.L., Goh J.C.H. (2009). Anterior Cruciate Ligament Regeneration Using Mesenchymal Stem Cells and Silk Scaffold in Large Animal Model. Biomaterials.

[71] Wang Y., Rudym D.D., Walsh A., Abrahamsen L., Kim H., Kim H.S., Kirker-head C., Kaplan D.L. (2008). In Vivo Degradation of Three-Dimensional Silk Fibroin Scaffolds. Biomaterials.

[72] Melke J., Midha S., Ghosh S., Ito K., Hofmann S. (2016). Silk Fibroin as Biomaterial for Bone Tissue Engineering. Acta Biomater..

[73] Johari N., Moroni L., Samadikuchaksaraei A. (2020). Tuning the Conformation and Mechanical Properties of Silk Fibroin Hydrogels. Eur. Polym. J..

[74] Grabska-Zielińska S., Sionkowska A., Coelho C.C., Grabska-zieli S., Monteiro F.J. (2020). Silk Fibroin/Collagen/Chitosan Scaffolds Cross-Linked by a Glyoxal Solution as Biomaterials toward Bone Tissue Regeneration. Materials.

[75] Eivazzadeh-keihan R., Ahmadpour F., Aghamirza H., Aliabadi M., Radinekiyan F., Maleki A., Madanchi H., Mahdavi M., Esmail A., Lanceros-m S. (2021). Pectin-Cellulose Hydrogel, Silk Fibroin and Magnesium Hydroxide Nanoparticles Hybrid Nanocomposites for Biomedical Applications. Int. J. Biol. Macromol..

[76] Chen T., Wen T., Dai N., Hsu S. (2021). Cryogel Hydrogel Biomaterials and Acupuncture Combined to Promote Diabetic Skin Wound Healing through Immunomodulation. Biomaterials.

[77] Hoon D., Tripathy N., Hun J., Eun J., Geun J., Dan K., Hum C., Khang G. (2017). Enhanced Osteogenesis of B-Tricalcium Phosphate Reinforced Silk Fibroin Scaffold for Bone Tissue Biofabrication. Int. J. Biol. Macromol..

[78] Chen Z., Zhong N., Wen J., Jia M., Guo Y., Shao Z., Zhao X., Accepted J. (2018). Porous Three-Dimensional Silk Fibroin Scaffolds for Tracheal Epithelial Regeneration in Vitro and in Vivo. ACS Biomater. Sci. Eng..

[79] Yan L.-P., Oliveira J.M., Oliveira A.L., Caridade S.G., Mano J.F., Reis R.L. (2012). Macro/Microporous Silk Fibroin Scaffolds with Potential for Articular Cartilage and Meniscus Tissue Engineering Applications. Acta Biomater..

[80] Vepari C., Kaplan D.L. (2007). Silk as a Biomaterial. Prog. Polym. Sci..

[81] Zhang H., Fang J., Ge H., Han L., Wang X., Hao Y., Han C., Dong L. (2013). Thermal, Mechanical, and Rheological Properties of Polylactide/Poly (1, 2-Propylene Glycol Adipate). Polym. Eng. Sci..

[82] Bunster G.F. (2016). Polyhydroxyalkanoates: Production and Use in Medicine. Encyclopedia of Biomedical Polymers and Polymeric Biomaterials.

[83] Guhados G., Wan W., Hutter J.L. (2005). Measurement of the Elastic Modulus of Single Bacterial Cellulose Fibers Using Atomic Force Microscopy. Am. Chem. Soc..

[84] Pogorelova N., Rogachev E., Dige I., Chernigova S., Nardin D. (2020). Bacterial Cellulose Nanocomposites: Morphology. Materials.

[85] Guo S., Dipietro L.A. (2010). Factors Affecting Wound Healing. Crit. Rev. Oral Biol. Med..

[86] Chen Z., Zhang Y., Zheng L., Zhang H., Shi H., Zhang X., Liu B. (2021). Mineralized Self-Assembled Silk Fibroin/Cellulose Interpenetrating Network Aerogel for Bone Tissue Engineering. Mater. Sci. Eng. C.

[87] Martınez-Mora C., Mrowiec A., Marı E., Alcaraz A., Jose F. (2012). Fibroin and Sericin from *Bombyx mori* Silk Stimulate Cell Migration through Upregulation and Phosphorylation Of. PLoS ONE.

[88] Nikam V.S., Punde D.S., Bhandari R.S. (2020). Silk Fibroin Nanofibers Enhance Cell Adhesion of Blood-Derived Fibroblast-like Cells—A Potential Application for Wound Healing. Indian J. Pharmacol..

[89] Gharehnazifam Z., Dolatabadi R., Baniassadi M., Shahsavari H., Kajbafzadeh A., Abrinia K., Baghani M. (2021). Computational Analysis of Vincristine Loaded Silk Fibroin Hydrogel for Sustained Drug Delivery Applications: Multiphysics Modeling and Experiments. Int. J. Pharm..

[90] Kwon G., Heo B., Kwon M.J., Kim I., Chu J., Kim B., Kim B., Park S.S. (2021). Effect of Silk Fibroin Biomaterial Coating on Cell Viability and Intestinal Adhesion of Probiotic Bacteria. J. Microbiol. Biotechnol..

[91] Lee O.J., Sultan M.T., Hong H., Lee Y.J., Lee J.S., Lee H., Kim S.H., Park C.H. (2020). Recent Advances in Fluorescent Silk Fibroin. Front. Mater..

[92] Baci G.-M., Cucu A.-A., Giurgiu A.-I., Muscă A.-S., Rațiu C.A., Bagameri L., Moise A.R., Bobiș O., Dezmirean D.S. (2022). Advances in Editing Silkworms (*Bombyx mori*) Genome by Using the CRISPR-Cas System. Insects.

[93] Nagano A., Tanioka Y., Sakurai N., Sezutsu H., Kuboyama N., Kiba H. (2011). Regeneration of the Femoral Epicondyle on Calcium-Binding Silk Scaffolds Developed Using Transgenic Silk Fibroin Produced by Transgenic Silkworm. Acta Biomater..

[94] Kuwana Y., Sezutsu H., Nakajima K.I., Tamada Y., Kojima K. (2014). High-Toughness Silk Produced by a Transgenic Silkworm Expressing Spider (*Araneus Ventricosus*) Dragline Silk Protein. PLoS ONE.

[95] Fujinaga D., Kohmura Y., Okamoto N., Kataoka H., Mizoguchi A. (2017). Insulin-like Growth Factor (IGF)-like Peptide and 20-Hydroxyecdysone Regulate the Growth and Development of the Male Genital Disk through Different Mechanisms in the Silkmoth, *Bombyx mori*. Insect Biochem. Mol. Biol..

[96] Wang F., Xu H., Wang Y., Wang R., Yuan L., Ding H., Song C., Ma S., Peng Z., Peng Z. (2014). Advanced Silk Material Spun by a Transgenic Silkworm Promotes Cell Proliferation for Biomedical Application. Acta Biomater..

[97] Wang M., Du Y., Huang H., Zhu Z., Du S., Chen S., Zhao H. (2019). Silk Fibroin Peptide Suppresses Proliferation and Induces Apoptosis and Cell Cycle Arrest in Human Lung Cancer Cells. Acta Pharmacol. Sin..

[98] Saviane A., Romoli O., Bozzato A., Freddi G., Cappelletti C., Rosini E., Cappellozza S., Tettamanti G. (2018). Intrinsic Antimicrobial Properties of Silk Spun by Genetically Modified Silkworm Strains. Transgenic Res..

[99] Li Z., Cao G., Xue R., Chengliang G. (2014). Construction of Transgenic Silkworm Spinning Antibacterial Silk with Fluorescence Construction of Transgenic Silkworm Spinning Antibacterial Silk with Fluorescence. Mol. Biol. Rep..

[100] Iizuka T., Sezutsu H., Tatematsu K., Kobayashi I., Yonemura N., Uchino K., Nakajima K., Kojima K., Takabayashi C., Machii H. (2013). Colored Fluorescent Silk Made by Transgenic Silkworms. Adv. J. Mater..

[101] Asakura T., Isozaki M., Saotome T., Tatematsu K., Sezutsu H., Kuwabara N., Nakazawa Y. (2014). Recombinant Silk Fibroin Incorporated Cell-Adhesive Sequences Produced by Transgenic Silkworm as a Possible Candidate for Use in Vascular Graft. J. Mater. Chem. B Mater. Biol. Med..

[102] Zhao S., Ye X., Wu M., Ruan J., Wang X., Tang X., Zhong B. (2021). Recombinant Silk Proteins with Additional Polyalanine Have Excellent Mechanical Properties. Int. J. Mol. Sci..

[103] Yanagisawa S., Zhu Z., Kobayashi I., Uchino K., Tamada Y., Tamura T., Asakura T. (2007). Improving Cell-Adhesive Properties of Recombinanant *Bombyx mori* Silk by Incorporation of Collagen or Fibronectin Derived Peptides Produced by Transgenic Silkworms. Biomacromolecules.

[104] Nguyen T.P., Nguyen Q.V., Nguyen V., Le T., Le Q.V. (2019). Silk Fibroin-Based Biomaterials for Biomedical. Polymers.

[105] Santos M.V., Paula K.T., Andrade M.B. De, Gomes E.M., Marques L.F., Ribeiro S.J.L., Mendonc C.R. (2020). Direct Femtosecond Laser Printing of Silk Fibroin Microstructures. Appl. Mater. Interfaces.

[106] Ho W., Jeong L., Il D., Hudson S. (2004). Effect of Chitosan on Morphology and Conformation of Electrospun Silk Fibroin Nanofibers. Polymer.

[107] Çalamak S., Erdo C., Özalp M., Ulubayram K. (2014). Silk Fibroin Based Antibacterial Bionanotextiles as Wound Dressing Materials. Mater. Sci. Eng. C.

[108] Ha S., Tonelli A.E., Hudson S.M., Carolina N. (2005). Structural Studies of *Bombyx mori* Silk Fibroin during Regeneration from Solutions and Wet Fiber Spinning. Biomacromolecules.

[109] Zhang F., Ming J., Dou H., Liu Z. (2014). Silk Dissolution and Regeneration at the Nanofibril Scale. J. Mater. Chem. B.

[110] Sato M., Nakazawa Y., Takahashi R., Tanaka K., Sata M., Aytemiz D., Asakura T. (2010). Small-Diameter Vascular Grafts of *Bombyx mori* Silk Fibroin Prepared by a Combination of Electrospinning and Sponge Coating. Mater. Lett..

[111] Aznar-cervantes S., Roca M.I., Martinez J.G., Meseguer-olmo L., Cenis J.L., Moraleda J.M., Otero T.F. (2012). Fabrication of Conductive Electrospun Silk Fibroin Scaffolds by Coating with Polypyrrole for Biomedical Applications. Bioelectrochemistry.

[112] Aznar-cervantes S.D., Vicente-cervantes D., Meseguer-olmo L., Cenis J.L., Lozano-pérez A.A. (2013). Influence of the Protocol Used for Fibroin Extraction on the Mechanical Properties and Fiber Sizes of Electrospun Silk Mats. Mater. Sci. Eng. C.

[113] Yamada H., Nakao H., Takasu Y., Tsubouchi K. (2001). Preparation of Undegraded Native Molecular Fibroin Solution from Silkworm Cocoons. Mater. Sci. Eng. C.

[114] Sofia S., Mccarthy M.B., Gronowicz G., Kaplan D.L., Al S.E.T. (2000). Functionalized Silk-Based Biomaterials for Bone Formation. J. Biomed. Mater. Res..

[115] Mathur A.B., Tonelli A., Rathke T., Hudson S. (1998). The Dissolution and Characterization of *Bombyx mori* Silk Fibroin in Calcium Nitrate-methanol.Pdf. Fiber Polym. Sci..

[116] Kunz W., Katharina H. (2018). Some Aspects of Green Solvents. Comptes Rendus Chim..

[117] Wang H., Zhang Y., Wei Z. (2021). Dissolution and Processing of Silk Fibroin for Materials Science. Crit. Rev. Biotechnol..

[118] Wöltje M., Kölbel A., Aibibu D., Cherif C. (2021). A Fast and Reliable Process to Fabricate Regenerated Silk Fibroin Solution from Degummed Silk in 4 Hours. Int. J. Mol. Sci..

[119] Freddi G., Pessina G., Tsukada M. (1999). Swelling and Dissolution of Silk Fibroin (*Bombyx mori*) in N -Methyl Morpholine N -Oxide. Biol. Macromol..

[120] Carissimi G., Baronio C.M., Montalbán M.G., Víllora G., Barth A. (2020). On the Secondary Structure of Silk Fibroin Nanoparticles Obtained Using Ionic Liquids: An Infrared Spectroscopy Study. Polymers.

[121] Garc M., Aznar-Cervantes S.D., Lozano-Perez A.A., Cenis L., Gloria V. (2015). Production of Silk Fibroin Nanoparticles Using Ionic Liquids and High-Power Ultrasounds. J. Appl. Polym. Sci..

[122] Phillips D.M., Drummy L.F., Conrady D.G., Fox D.M., Naik R.R., Stone M.O., Trulove P.C., Long H.C. De, Mantz R.A. (2004). Dissolution and Regeneration of *Bombyx mori* Silk Fibroin Using Ionic Liquids. J. Am. Chem. Soc..

[123] Ajisawa A. (1997). Dissolution Aqueous of Silk Fibroin with Calciumchloride/Ethanol Solution. J. Seric. Sci. Jpn..

[124] Reizabal A., Costa C.M., Saiz P.G., Gonzalez B., Perez-Alvarez L., Luis R.F. de, Garcia A., Vilas-Vilela J., Lanceros-Mendez S. (2021). Processing Strategies to Obtain Highly Porous Silk Fibroin Structures with Tailored Microstructure and Molecular Characteristics and Their Applicability in Water Remediation. J. Hazard. Mater..

[125] Bae S. Bin, Kim M.H., Park W.H. (2020). Electrospinning and Dual Crosslinking of Water-Soluble Silk Fibroin Modified with Glycidyl Methacrylate. Polym. Degrad. Stab..

[126] Mosher C.Z., Brudnicki P.A.P., Gong Z., Childs H.R., Lee S.W., Antrobus R.M., Fang E.C., Schiros T.N., Lu H.H. (2021). Green Electrospinning for Biomaterials and Biofabrication. Biofabrication.

[127] Li X., Fan Q., Zhang Q., Yan S., You R. (2020). Freezing-Induced Silk I Crystallization of Silk Fibroin. R. Soc. Chem..

[128] Yang X., Wang X., Yu F., Ma L., Pan X., Luo G., Lin S., Mo X., Wang H. (2016). Hyaluronic Acid/EDC/NHS-Crosslinked Green Electrospun Silk Fibroin Nanofibrous Scaffolds for Tissue Engineering. R. Soc. Chem..

[129] Fei X., Jia M., Du X., Yang Y., Zhang R., Shao Z., Zhao X. (2013). Green Synthesis of Silk Fibroin-Silver Nanoparticle Composites with Effective Antibacterial and Biofilm-Disrupting Properties. Biomacromolecules.

[130] Raho R., Nguyen N., Zhang N., Jiang W., Sannino A., Liu H., Pollini M., Paladini F. (2020). Photo-Assisted Green Synthesis of Silver Doped Silk Fibroin/Carboxymethyl Cellulose Nanocomposite Hydrogels for Biomedical Applications. Mater. Sci. Eng. C.

[131] El-Sheikh M.A., El-Rafie S.M., Abdel-Halim E.S., El-Rafie M.H. (2013). Green Synthesis of Hydroxyethyl Cellulose-Stabilized Silver Nanoparticles. J. Polym..

[132] Altman G.H., Diaz F., Jakuba C., Calabro T., Horan R.L., Chen J., Lu H., Richmond J., Kaplan D.L. (2003). Silk-Based Biomaterials. Biomaterials.

[133] Kamalathevan P., Ooi P.S., Loo Y.L. (2018). Silk-Based Biomaterials in Cutaneous Wound Healing: A Systematic Review. Adv. Ski. Wound Care.

[134] Cheng G., Wang X., Tao S., Xia J., Xu S. (2015). Differences in Regenerated Silk Fibroin Prepared with Different Solvent Systems: From Structures to Conformational Changes. J. Appl. Polym. Sci..

[135] Volkov V., Ferreira A.V., Cavaco-paulo A. (2015). On the Routines of Wild-Type Silk Fibroin Processing Toward Silk-Inspired Materials: A Review. Macromol. Mater. Eng..

[136] Jin S.C., Baek H.S., Woo Y.I., Lee M.H., Kim J., Park J., Park Y.H., Lee S.J. (2009). Beneficial Effects of Microwave-Induced Argon Plasma Treatment on Cellular Behaviors of Articular Chondrocytes onto Nanofibrous Silk Fibroin Mesh. Macromol. Res..

[137] Gu J., Yang X., Zhu H. (2002). Surface Sulfonation of Silk Fibroin Film by Plasma Treatment and in Vitro Antithrombogenicity Study. Mater. Sci. Eng. C.

[138] Shang K., Rnjak-kovacina J., Tao H., Kaplan D.L., Lin Y., Hayden R.S. (2013). Accelerated In Vitro Degradation of Optically Clear Low b -Sheet Silk Films by Enzyme-Mediated Pretreatment. Transl. Vis. Sci. Technol..

[139] Wang L., Nemoto R., Senna M. (2004). Three-Dimensional Porous Network Structure Developed in Hydroxyapatite-Based Nanocomposites Containing Enzyme Pretreated Silk Fibroin. J. Nanopart. Res..

[140] Li M., Ogiso M., Minoura N. (2003). Enzymatic Degradation Behavior of Porous Silk Fibroin Sheets. Biomaterials.

[141] Wang P., Zhou Y., Cui L., Yuan J., Wang Q., Fan X., Ding Y. (2014). Enzymatic Grafting of Lactoferrin onto Silk Fibroins for Antibacterial Functionalization. Fibers Polym..

[142] Chi R., Cheung F., Ng T.B., Wong J.H., Chan W.Y. (2015). Chitosan: An Update on Potential Biomedical and Pharmaceutical Applications. Mar. Drugs.

[143] Li X., Li B., Wang X., Zhang S. (2014). Development of a Silk Fibroin/HTCC/PVA Sponge for Chronic Wound Dressing. J. Bioact. Compat. Polym. Biomed. Appl..

[144] Karahaliloğlu Z. (2018). Curcumin-Loaded Silk Fibroin e-Gel Scaffolds for Wound Healing Applications. Mater. Technol..

[145] Thangavel P., Ramachandran B., Kannan R., Muthuvijayan V. (2016). Biomimetic Hydrogel Loaded with Silk and L -Proline for Tissue Engineering and Wound Healing Applications. Soc. Biomater..

[146] Luo Z., Jiang L., Xu Y., Li H., Xu W., Wu S., Wang Y., Tang Z., Lv Y., Yang L. (2015). Biomaterials Mechano Growth Factor (MGF) and Transforming Growth Factor (TGF)—B3 Functionalized Silk Scaffolds Enhance Articular Hyaline Cartilage Regeneration in Rabbit Model. Biomaterials.

[147] Mehrabani M.G., Karimian R., Rakhshaei R., Pakdel F., Eslami H., Fakhrzadeh V., Rahimi M., Salehi R., Kafil H.S. (2018). Chitin/Silk Fibroin/TiO_2_ Bio-Nanocomposite as a Biocompatible Wound Dressing Bandage with Strong Antimicrobial Activity. Biol. Macromol..

[148] Patil P.P., Meshram J.V., Bohara R.A. (2018). ZnO Nanoparticle-Embedded Silk Fibroin–Polyvinyl Alcohol Composite Film: A Potential Dressing Material for Infected Wounds. R. Soc. Chem..

[149] Li X., Liu Y., Zhang J., You R., Qu J., Li M. (2017). Functionalized Silk Fibroin Dressing with Topical Bioactive Insulin Release for Accelerated Chronic Wound Healing. Mater. Sci. Eng. C.

[150] Hrynyk M., Neufeld R.J. (2014). Insulin and Wound Healing. Burns.

[151] Wang Y., Kim U., Blasioli D.J., Kim H., Kaplan D.L. (2005). In Vitro Cartilage Tissue Engineering with 3D Porous Aqueous-Derived Silk Scaffolds and Mesenchymal Stem Cells. Biomaterials.

[152] Hee S., Been Y., Kyu Y., Jin Y., Sang H., Sultan T., Min J., Seung J., Joo O., Hong H. (2020). Biomaterials 4D-Bioprinted Silk Hydrogels for Tissue Engineering. Biomaterials.

[153] Mallepally R.R., Marin M.A., Surampudi V., Subia B., Rao R.R. (2015). Silk Fibroin Aerogels: Potential Scaffolds for Tissue Engineering Applications. Biomed. Mater..

[154] Nazarov R., Jin H., Kaplan D.L. (2004). Porous 3-D Scaffolds from Regenerated Silk Fibroin. Biomacromolecules.

[155] Pacheco S.M., Eiji G., Almeida L. De, Santos P., Agostini M., Moraes D. (2020). Silk Fibroin/Chitosan/Alginate Multilayer Membranes as a System for Controlled Drug Release in Wound Healing. Int. J. Biol. Macromol..

[156] Harkin D.G., George K.A., Madden P.W., Schwab I.R., Hutmacher D.W., Chirila T.V. (2011). Biomaterials Silk Fibroin in Ocular Tissue Reconstruction. Biomaterials.

[157] Calonge M., Nieto-Miguel T., Mata A. De, Galindo S., Herreras J.M., Marina L. (2021). Goals and Challenges of Stem Cell-Based Therapy for Corneal Blindness Due to Limbal Deficiency. Pharmaceutics.

[158] Lee M.C., Kim D., Lee O.J., Kim J., Ju H.W., Lee J.M., Moon B.M., Park H.J., Kim D.W., Kim S.H. (2016). Fabrication of Silk Fibroin Film Using Centrifugal Casting Technique for Corneal Tissue Engineering. Soc. Biomater..

[159] Liu J., Lawrence B.D., Liu A., Schwab I.R., Oliveira L.A., Rosenblatt M.I. (2012). Silk Fibroin as a Biomaterial Substrate for Corneal Epithelial Cell Sheet Generation. Investig. Ophthalmol. Vis. Sci..

[160] Pellegrini G., Dellambra E., Golisano O., Martinelli E., Fantozzi I., Bondanza S., Ponzin D., Mckeon F., Luca M. De. (2001). P63 Identifies Keratinocyte Stem Cells. Proc. Natl. Acad. Sci. USA.

[161] Schermer A., Galvin S. (1986). Differentiation-Related Expression of a Major 64K Corneal Keratin In Vivo and In Culture Suggests Limbal Location of Corneal Epithelial Stem Cells. J. Cell Biol..

[162] Lawrence B.D., Marchant J.K., Pindrus M.A., Omenetto F.G., Kaplan D.L. (2009). Biomaterials Silk Film Biomaterials for Cornea Tissue Engineering. Biomaterials.

[163] Malliappan P., Alp A., Burcu S., Demir E., Cetinel S. (2022). Bone Tissue Engineering: Anionic Polysaccharides as Promising Scaffolds. Carbohydr. Polym..

[164] Sk S. (2019). Fracture Non-Union: A Review of Clinical Challenges and Future Research Needs. Malays. Orthop. J..

[165] Gillman C.E., Jayasuriya A.C. (2021). FDA-Approved Bone Grafts and Bone Graft Substitute Devices in Bone Regeneration. Mater. Sci. Eng. C.

[166] Zhao Z., Ma X., Ma J., Kang J., Zhang Y., Guo Y. (2022). Sustained Release of Naringin from Silk- Fibroin-Nanohydroxyapatite Scaffold for the Enhancement of Bone Regeneration. Mater. Today Bio.

[167] Meinel L., Fajardo R., Hofmann S., Langer R., Chen J., Snyder B., Vunjak-novakovic G., Kaplan D. (2005). Silk Implants for the Healing of Critical Size Bone Defects. Bone.

[168] Zhao J., Zhang Z., Wang S., Sun X., Zhang X., Chen J., Kaplan D.L., Jiang X. (2009). Apatite-Coated Silk Fibroin Scaffolds to Healing Mandibular Border Defects in Canines. Bone.

[169] Hirose M., Hamada K., Tanaka T. (2006). Nano-Scaled Hydroxyapatite/Silk Fibroin Composites as Mesenchymal Cell Culture Scaffolds. Key Eng. Mater..

[170] Cui B., Liang L., Lu X., Weng J. (2007). Fabricating HYDROXYAPATITE—SIlk Fibroin Nanocomposite by Bone Bionics. Key Eng. Mater..

[171] Wen G., Wang J., Li M., Meng X. (2007). Study on Tissue Engineering Scaffolds of Silk Fibroin-Chitosan/Nano-Hydroxyapatite Composite. Key Eng. Mater..

[172] Sun B.K., Siprashvili Z., Khavari P.A. (2014). Advances in Skin Grafting and Treatment of Cutaneous Wounds. Science.

[173] Pra I.D.A.L., Chiarini A., Boschi A., Freddi G., Armato U. (2006). Novel Dermo-Epidermal Equivalents on Silk Fibroin-Based Formic Acid-Crosslinked Three-Dimensional Nonwoven Devices with Prospective Applications in Human Tissue Engineering/Regeneration/Repair. Int. J. Mol. Med..

[174] Luangbudnark W., Viyoch J., Laupattarakasem W., Surakunprapha P., Laupattarakasem P. (2012). Properties and Biocompatibility of Chitosan and Silk Fibroin Blend Films for Application in Skin Tissue Engineering. Sci. World J..

[175] Bhardwaj N., Sow W.T., Devi D., Ng K.W., Mandal B.B., Cho N.-J. (2014). Insight Statement for “Silk Fibroin-Keratin Based 3D Scaffolds as a Dermal Substitute for Skin Repair and Regeneration. Integr. Biol..

[176] Lyu J., Kaur M., Dibble K.E., Connor A.E. (2022). A National Study of Alcohol Consumption Patterns among Population-Based U. S. Cancer Survivors Compared with Cancer-Free Individuals. Cancer Epidemiol..

[177] Desforges A.D., Hebert C.M., Spence A.L., Reid B., Dhaibar A., Cruz-topete D., Cornett E.M., David A., Urits I., Viswanath O. (2022). Treatment and Diagnosis of Chemotherapy-Induced Peripheral Neuropathy: An Update. Biomed. Pharmacother..

[178] Ma D., Wang Y., Dai W. (2018). Silk Fibroin-Based Biomaterials for Musculoskeletal Tissue Engineering. Mater. Sci. Eng. C.

[179] Ma Y., Canup B.S.B., Tong X., Dai F., Xiao B., Wang J. (2020). Multi-Responsive Silk Fibroin-Based Nanoparticles for Drug Delivery. Front. Chem..

[180] Sun N., Lei R., Xu J., Kundu S.C. (2019). Fabricated Porous Silk Fibroin Particles for PH- Responsive Drug Delivery and Targeting of Tumor Cells. J. Mater. Sci..

[181] Saqr A. Al, Ud S., Wani D., Gangadharappa H.V., Aldawsari M.F., Khafagy E., Lila A.S.A. (2021). Enhanced Cytotoxic Activity of Docetaxel-Loaded Silk Fibroin Nanoparticles against Breast Cancer Cells. Polymers.

[182] Mottaghitalab F., Kiani M., Farokhi M., Kundu S.C., Reis R.L., Gholami M., Bardania H., Dinarvand R., Geramifar P., Beiki D. (2017). Targeted Delivery System Based on Gemcitabine Loaded Silk Fibroin Nanoparticles for Lung Cancer Therapy Targeted Delivery System Based on Gemcitabine Loaded Silk Fibroin Nanoparticles for Lung Cancer Therapy. Appl. Mater. Interfaces.

[183] Moin A., Wani S.U.D., Osmadi R.A., Lila A.S.A., Khafagy E.-S., Arab H.H., Gangaharappa H.V., Allam A.N. (2021). Formulation, Characterization, and Cellular Toxicity Assessment of Tamoxifen-Loaded Silk Fibroin Nanoparticles in Breast Cancer. Drug Deliv..

[184] Lu X., Sun Y., Han M., Chen D., Wang A., Sun K. (2022). Silk Fibroin Double-Layer Microneedles for the Encapsulation and Controlled Release of Triptorelin. Int. J. Pharm..

[185] Dondajewska E., Juzwa W., Mackiewicz A., Dams- H. (2018). Heterotypic Breast Cancer Model Based on a Silk Fibroin Scaffold to Study the Tumor Microenvironment. Oncotarget.

[186] Li J., Zhou Y., Chen W., Yuan Z., You B., Liu Y., Yang S., Li F., Qu C., Zhang X. (2018). A Novel 3D in Vitro Tumor Model Based on Silk Fibroin/Chitosan Sca Ff Olds to Mimic the Tumor Microenvironment. Appl. Mater. Interfaces.

[187] Qian K., Song Y., Yan X., Dong L., Xue J., Xu Y., Wang B., Cao B., Hou Q., Peng W. (2020). Biomaterials Injectable Ferrimagnetic Silk Fibroin Hydrogel for Magnetic Hyperthermia Ablation of Deep Tumor. Biomaterials.

[188] Mukhopadhyay S.K., Naskar D., Bhattacharjee P., Mishra A., Kundu S.C., Dey S. (2017). Silk Fibroin-Thelebolan Matrix: A Promising Chemopreventive Scaffold for Soft Tissue Cancer. Colloids Surf. B Biointerfaces.

[189] Pandey V., Haider T., Chandak A.R., Chakraborty A., Banerjee S., Soni V. (2020). Technetium Labeled Doxorubicin Loaded Silk Fibroin Nanoparticles: Optimization, Characterization and in Vitro Evaluation. J. Drug Deliv. Sci. Technol..

[190] Anas M., Hadianamrei R., Sun W., Xu D., Brown S., Zhao X. (2021). Stiffness-Tuneable Nanocarriers for Controlled Delivery of ASC-J9 into Colorectal Cancer Cells. J. Colloid Interface Sci..

[191] Talukdar S., Kundu S.C. (2013). Engineered 3D Silk-Based Metastasis Models: Interactions Between Human Breast Adenocarcinoma, Mesenchymal Stem Cells and Osteoblast-Like Cells. Adv. Funct. Mater..

[192] Ding B., Wahid M.A., Zhijun W., Chen X., Arvind T., Sunil P., Wang J. (2017). Triptolide and Celastrol Loaded Silk Fibroin Nanoparticles Show Synergistic Effect against Human Pancreatic Cancer Cells. Nanoscale.

[193] Toan D., Saelim N., Tiyaboonchai W. (2019). Alpha Mangostin Loaded Crosslinked Silk Fibroin-Based Nanoparticles for Cancer Chemotherapy. Colloids Surf. B Biointerfaces.

[194] Yucel T., Lovett M.L., Giangregorio R., Coonahan E., Kaplan D.L. (2014). Silk Fibroin Rods for Sustained Delivery of Breast Cancer Therapeutics. Biomaterials.

[195] Zhang X., Huang Y., Song H., Canup B.S.B., Gou S. (2020). Inhibition of Growth and Lung Metastasis of Breast Cancer by Tumor-Homing Triple-Bioresponsive Nanotherapeutics. J. Control. Release.

[196] Yao Q., Lan Q., Jiang X., Du C., Zhai Y., Shen X., Xu H. (2020). Bioinspired Biliverdin/Silk Fibroin Hydrogel for Antiglioma Photothermal Therapy and Wound Healing. Theranostics.

[197] Yu S., Yang W., Chen S., Chen M., Liu Y., Shao Z., Chen X. (2014). Floxuridine-Loaded Silk Fibroin Nanospheres. RSC Adv..

[198] Mishra D., Iyyanki T.S., Hubenak J.R., Zhang Q., Mathur A.B. (2017). Silk Fibroin Nanoparticles and Cancer Therapy. Nanotechnology in Cancer.

